# Impact of the nisin modification machinery on the transport kinetics of NisT

**DOI:** 10.1038/s41598-020-69225-2

**Published:** 2020-07-23

**Authors:** Marcel Lagedroste, Jens Reiners, Sander H. J. Smits, Lutz Schmitt

**Affiliations:** 1 Institute of Biochemistry, Heinrich Heine University Düsseldorf, Universitätsstr. 1, 40225 Düsseldorf, Germany; 2 Present Address: Center for Structural Studies, Heinrich Heine University Düsseldorf, Universitätsstr. 1, 40225 Düsseldorf, Germany

**Keywords:** Membrane proteins, Biochemistry, Enzymes, Proteins

## Abstract

Lanthipeptides are ribosomally synthesized and post-translationally modified peptides containing dehydrated amino acids and (methyl-)lanthionine rings. One of the best-studied examples is nisin produced by *Lactococcus lactis*. Nisin is synthesized as a precursor peptide comprising of an N-terminal leader peptide and a C-terminal core peptide. Amongst others, the leader peptide is crucial for enzyme recognition and acts as a secretion signal for the ABC transporter NisT that secretes nisin in a proposed channeling mechanism. Here, we present an in vivo secretion analysis of this process in the presence and absence of the nisin maturation machinery, consisting of the dehydratase NisB and the cyclase NisC. Our determined apparent secretion rates of NisT show how NisB and NisC modulate the transport kinetics of NisA. Additional in vitro studies of the detergent-solubilized NisT revealed how these enzymes and the substrates again influence the activity of transporter. In summary, this study highlights the pivotal role of NisB for NisT in the secretion process.

## Introduction

Many natural products (NP) produced as secondary metabolites by microorganisms can be used as pharmaceuticals (e.g. as anticancer, antibacterial or antiviral drugs)^1^. One class of these NPs are ribosomally synthesized and post-translationally modified peptides (RiPPs). The RiPP family of lanthipeptides, especially those with antimicrobial activity (lantibiotics), is gaining interest as a potential alternative for antibiotics to treat harmful multidrug resistance strains such as methicillin-resistant *Staphylococcus aureus* or vancomycin-resistant *Enterococci*^2,3^.

Lanthipeptides (LanA) are produced as precursor peptides with an N-terminal leader peptide (LP) and a C-terminal core peptide (CP)^4^. The LP serves as a signal sequence and recognition site for the modification enzymes and the export protein^5–8^. Furthermore, the LP keeps the modified peptide (mLanA) inactive in the cytosol^9^, while the post-translational modifications (PTM) are installed within the CP and not found in the LP^10^. These PTM’s are unusual amino acids such as didehydroalanine (Dha), didehydrobutyrine (Dhb) or (methyl-)lanthionine ((Me)Lan)^11,12^.

**Figure 1:**
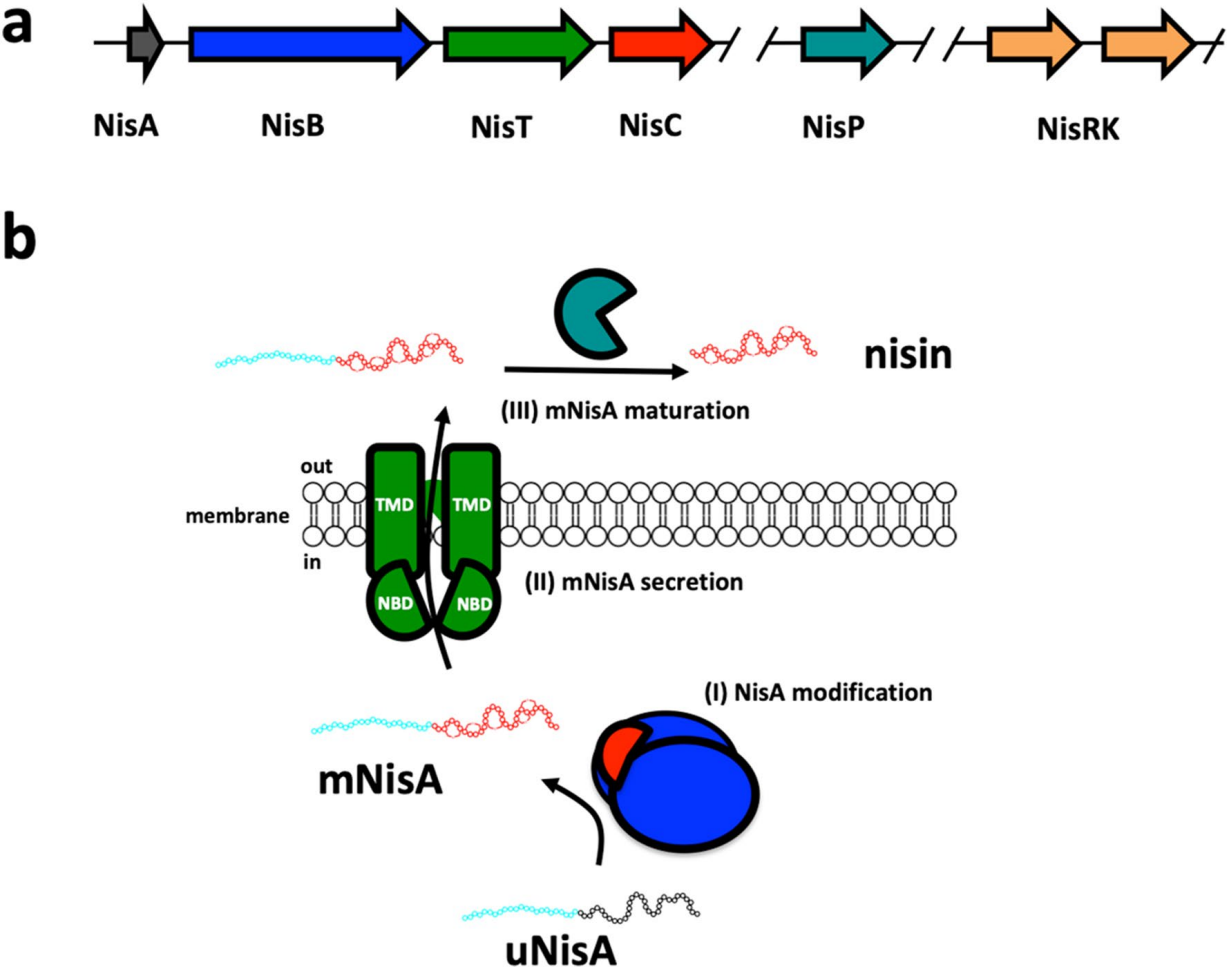
Scheme of nisin modification and secretion system. (**a**) The lanthipeptide nisin (NisA, grey) operon encodes for the modification and secretion enzymes. (**b**) The enzyme NisB (blue) catalyzes the dehydration reaction of unmodified NisA (uNisA), whereas NisC (red) catalyzes the thioether ring formation resulting in modified NisA (mNisA). The ABC transporter NisT (green) translocates mNisA across the membrane to the exterior. Finally, the mature peptide is processed by the serine protease NisP (turquoise) and active nisin is released. The two-component system (TCS) consisting of NisR and NisK (orange) is controlling the expression of these proteins. Please note, that the operon is partial represented and shows only proteins responsible for nisin maturation and secretion.

Nisin is produced by the Gram-positive bacterium *Lactococcus lactis* as a precursor peptide (NisA), where the genes for modification, secretion and maturation enzymes are located on one operon (Fig. 1a)^8^. First, the ribosomally synthesized NisA is modified by the modification enzymes NisB and NisC (Fig. 1b, I). Within the unmodified NisA serine and threonine residues are dehydrated by the dehydratase NisB via a tRNA-depended glutamylation and elimination reaction to Dha and Dhb residues^13–15^. Subsequently, the dehydrated amino acid are coupled to neighboring cysteine residues via a Michael-like addition catalyzed stereo- and regio-specifically by the cyclase NisC^16–18^. The reaction of both enzymes follows an alternating mode with a N- to C-terminus directionality yielding (Me)Lan residues^19,20^. Next, the exporter protein NisT secretes the modified NisA (mNisA) to the exterior (Fig. 1b, II)^21^. Finally, the LP is cleaved by the extracellularly located serine protease NisP and active nisin is released (Fig. 1b, III)^10^.

The lanthipeptide exporters (LanT) belong to the superfamily of ABC transporters, which are found in all kingdoms of life^22^. Bacterial ABC transporters are composed of mainly two domains^23^. One domain is the transmembrane domain (TMD) important for substrate translocation. The other domain is the nucleotide-binding domain (NBD), which binds and hydrolyses ATP to energize conformational changes used for substrate translocation. Some lanthipeptide exporters harbor an additional domain, a C39 peptidase domain. This domain is classified as a bacteriocin-processing endopeptidase^24^ and we term this subfamily of transporters LanT_C39P_.

All known lanthipeptide exporters function as dimers and translocation of the lanthipeptide is LP-dependent^5,25–27^. For some LanT/ Lan_C39P_T proteins it is proposed that the exporter and modification proteins assemble a multimeric enzyme complex at the membrane, which translocate the substrate to the exterior (e.g. nisin, subtilin and nukacin ISK-1)^28–30^.

In 2004, it was shown that the secretion of nisin without the modification enzymes NisB, NisC and the protease NisP was possible^5^. In vivo studies on the secretion process expanded the knowledge on the nisin modification and secretion system^31–34^. According to these studies the observed high secretion efficiency of NisA by NisBTC was explained by a ‘channeling mechanism’^19,35^. Other studies focused on the application of the nisin modification machinery to produce nisin variants or lantibiotics and secrete them by NisT^36–39^. Despite the in vivo analysis of NisA secretion, a first analysis with labeled precursor peptides was performed and gave first insights into the kinetics of secretion^35^. Nevertheless, a systematic and quantitative analysis of the secretion mechanism by determining kinetic parameters for NisA translocation by NisT is still required.

In our study, we performed an in vivo and in vitro characterization of NisT to shed light on the secretion mechanism of NisA. We determined the kinetic parameters of NisA secretion by analyzing the supernatant of NisA secreting *L. lactis* strains via reverse-phase (RP-)HPLC. The resulting apparent secretion rate (NisA·NisT^−1^·min^−1^) of NisT was compared with the rate of the NisBTC system and demonstrated a large enhancement in the presence of the nisin modification machinery. The in vitro characterization of NisT is the first study revealing insights into the specific activity of a LanT lanthipeptide transporter and its modification enzymes as well as its substrate. In conclusion, we could demonstrate a direct enhancement of the secretion by the maturation enzymes and a bridging function of the dehydratase NisB for the interaction of NisC and NisT.

## Results

### In vivo secretion assay of NisA

**Figure 2:**
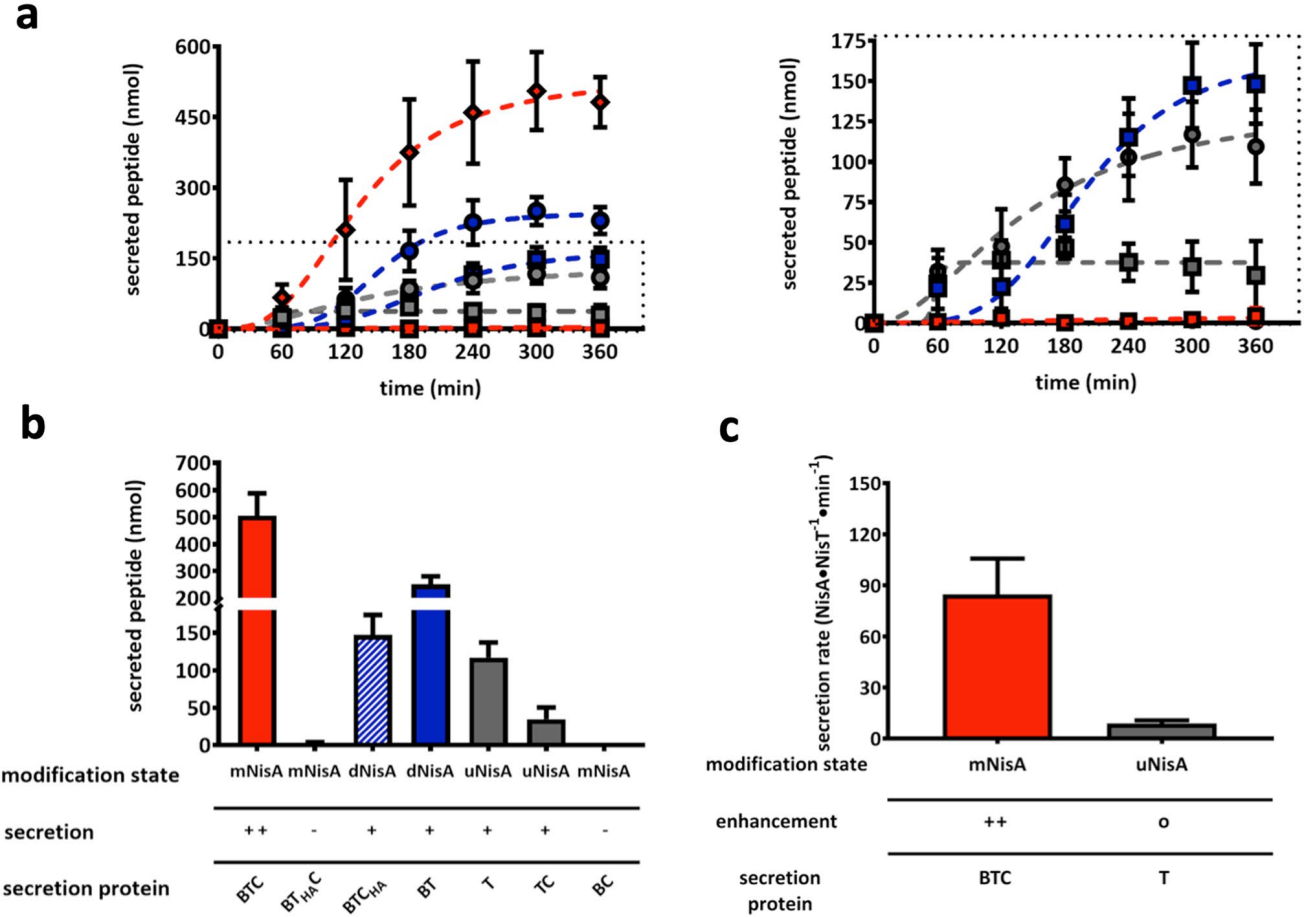
In vivo secretion assay of different *L. lactis* NZ9000 strains. (**a**) The supernatants of NisA secreting *L. lactis* NZ9000 strains was analyzed by RP-HPLC and the amount of NisA were determined. Amounts of secreted peptides (nmol) are plotted against time (min) and the resulting curves were fitted by an allosteric sigmoidal fit. Modified NisA (mNisA, red) was secreted by strain NZ9000BTC (red rhomb) and can be precluded by *nisT* deletion (strain NZ9000BC, clear dot) or an ATPase deficient mutant (NZ9000BT_H551A_C, red square). Dehydrated NisA (dNisA, blue) was secreted by strains NZ9000BTC_H331A_ (blue square) and NZ9000BT (blue dots), whereas unmodified NisA (uNisA, grey) was secreted by the strains NZ9000T (grey dots) and NZ9000TC (grey square). Dashed square shows a zoom-in on strains with lower secretion level. (**b**) The kinetic parameter of V_max_ (nmol) of secreted peptides was plotted as bars against the various secretion systems. (**c**) The secretion rate of NisA molecules per NisT molecule was plotted against time (min) and fitted by linear regression. The slope represented the secretion rate of NisA·NisT^−1^·min^−1^ for the strains NZ9000BTC and NZ9000T. All data represent secretion experiments from at least five different transformants and are represented as means ± s.d. (n = 5).  + + : WT secretion; o: low secretion; −: no secretion.

To obtain further insights into the mechanism of lanthipeptide secretion, the NisA secretion level of the *L. lactis* strain NZ9000 was investigated in the presence and absence of the modification machinery. Here, the well-known nisin secretion and maturation system^19,31,35,37^ was used to establish an in vivo secretion assay, where the supernatants were employed to determine the secretion level of NisA peptide via RP-HPLC analysis. In our study, we used the strain *L. lactis* NZ9000BTC (Table S1) producing fully modified NisA (mNisA) that is secreted by NisT. The secretion of other modification states of NisA can be analyzed by deletions of one of the modification enzymes or by creating inactive mutants. The mutation NisC_H331A_ (strain NZ9000BTC_H331A_) or the deletion of NisC (strain NZ9000BT) resulted in the secretion of dehydrated NisA (dNisA). Deletion of NisB (strain NZ9000TC) or NisB and NisC (strain NZ9000T) resulted in the secretion of unmodified NisA (uNisA). The deletion of NisT (strain NZ9000BC) or the mutation of the histidine of the H-loop in the NBD of NisT (strain NZ9000BT_H551A_C) totally abolished NisA secretion (Fig. 2). The latter two strains were defined as the background of the secretion analysis, in which NisA is expressed but not secreted. For the secretion analysis, all *L. lactis* strains were grown in minimal medium at 30 °C and after induction samples of supernatants were analyzed every hour (0–6 h). Subsequently, the supernatant was analyzed by RP-HPLC (Fig. 2, Figure S1) and the peak area of NisA peptides was determined to calculate the amount of peptide. The amount of secreted peptide was plotted against time and a non-linear fitting was applied to determine V_max_ (maximal amount of peptide; in nmol) and K_0.5_ (time point of 50% secreted peptide in min). These kinetic parameters allowed a direct comparison of the secretion efficiency of the different *L. lactis* strains employed in this study.

Strain NZ9000BTC had a V_max_ value of 534 ± 44 nmol and a K_0.5_ of 134 ± 12 min. It secreted NisA most efficiently in comparison to the other strains (Fig. 2, Table S3). In the cytoplasmic fraction of this strain only a small amount of mNisA was detected by Western blot at hours 2, 3 and 4 after induction (Figure S2a). This finding is similar to the previously published data for the nisin secretion and modification system^35^.

Mutation of the H-loop at position 551 to alanine in the NBD of the ABC transporter NisT abolished the secretion of mNisA and no peptide was detected in the supernatant (Fig. 2, Figure S2c). The same result was observed for the strain NZ9000BC (Figure S2g). Here, high amounts of mNisA were detected in the cytoplasmic fraction of the strains NZ9000BT_H551A_C and NZ9000BC (Figure S1c/g). Thus, *nisT* deletion and the H-loop mutation both abolished mNisA secretion.

Deletion of *nisC* (strain NZ9000BT) resulted in a lower secretion efficiency of dNisA and the V_max_ value of 247 ± 15 nmol and a K_0.5_ of 152 ± 9 min (Fig. 2, Table S3). The amount of secreted dNisA was reduced by a factor of 2.2 in comparison to mNisA (strain NZ9000BTC). Interestingly, the mutation of the catalytic histidine residue (H331) to alanine^40^ reduced the V_max_ value (168 ± 16 nmol) by factor of 3.2. The K_0.5_ value increased to 200 ± 16 min (Fig. 2). The analysis of the cytoplasmic fraction of the strain NZ9000BTC_H331A_ showed a higher amount of dNisA inside the cell, which only slowly decreased over the time (Figure S2b). Slightly lower amounts of dNisA were observed in the cytoplasmic fraction of NZ9000BT (Figure S2d).

Strain NZ9000T, which was obtained after the deletion of *nisB* and *nisC*, had a slightly reduced V_max_ value of 137 ± 30 nmol with a K_0.5_ of 144 ± 41 min (Fig. 2, Table S3). The secretion of uNisA was reduced by a factor of 3.9 compared to strain NZ9000BTC. The lowest amount of secreted peptide was determined in the supernatant of strain NZ9000TC with a V_max_ value of 38 ± 8 nmol. Here, a higher amount of uNisA was detected in the cytoplasmic fraction, whereas no peptide was observed in strain NZ9000T.

In all strains, NisB, NisC and NisT were detected in their corresponding fraction (cytoplasmic or membrane) (Figure S2a–g). The proteins NisB, NisC/NisC_H331A_ and NisA were observed in the cytoplasmic fractions (except uNisA from the cytoplasm of NZ9000T). NisT was detected by Western blot in the membrane fraction of all strains (Figure S2a–g). In the case of mNisA expressing strains (NZ9000BTC, NZ9000BT_H551A_C, and NZ9000BC) all proteins were detected by Western blot even at time point zero.

### Determination of the apparent secretion rate of mNisA

We measured the apparent secretion rate (V_S app._) by an in vivo secretion assay. First, the amount of secreted mNisA at different time points was determined and plotted as nmol mNisA against time (Fig. 2). Second, the amount of the ABC transporter NisT was obtained by analyzing the membrane fraction of strains NZ9000BTC and NZ9000T at each time point by Western blot (Figure S3a,b). Here, known concentrations of purified NBD protein were used as a standard to determine the amount of NisT in pmol. Combining these data, the secretion rate of NisA molecules per NisT molecules was calculated. The plot of nmol·NisA·NisT^−1^ against time (min) was fitted by linear regression (Figure S3c). The slope of the linear regression corresponds to V_S app._ of NisA·NisT^−1^·min^−1^, which is 84.8 ± 21 for the strain NZ9000BTC (Fig. 2c, Figure S3c). A strongly reduced secretion rate was determined for the strain NZ9000T (8.8 ± 1.8) as described qualitatively previously^35^. The determined secretion rate thus clearly demonstrated an enhancement in the presence of the modification enzymes NisB and NisC.

### Purification and basal ATPase activity of NisT, a class I lanthipeptide transporter

**Figure 3:**
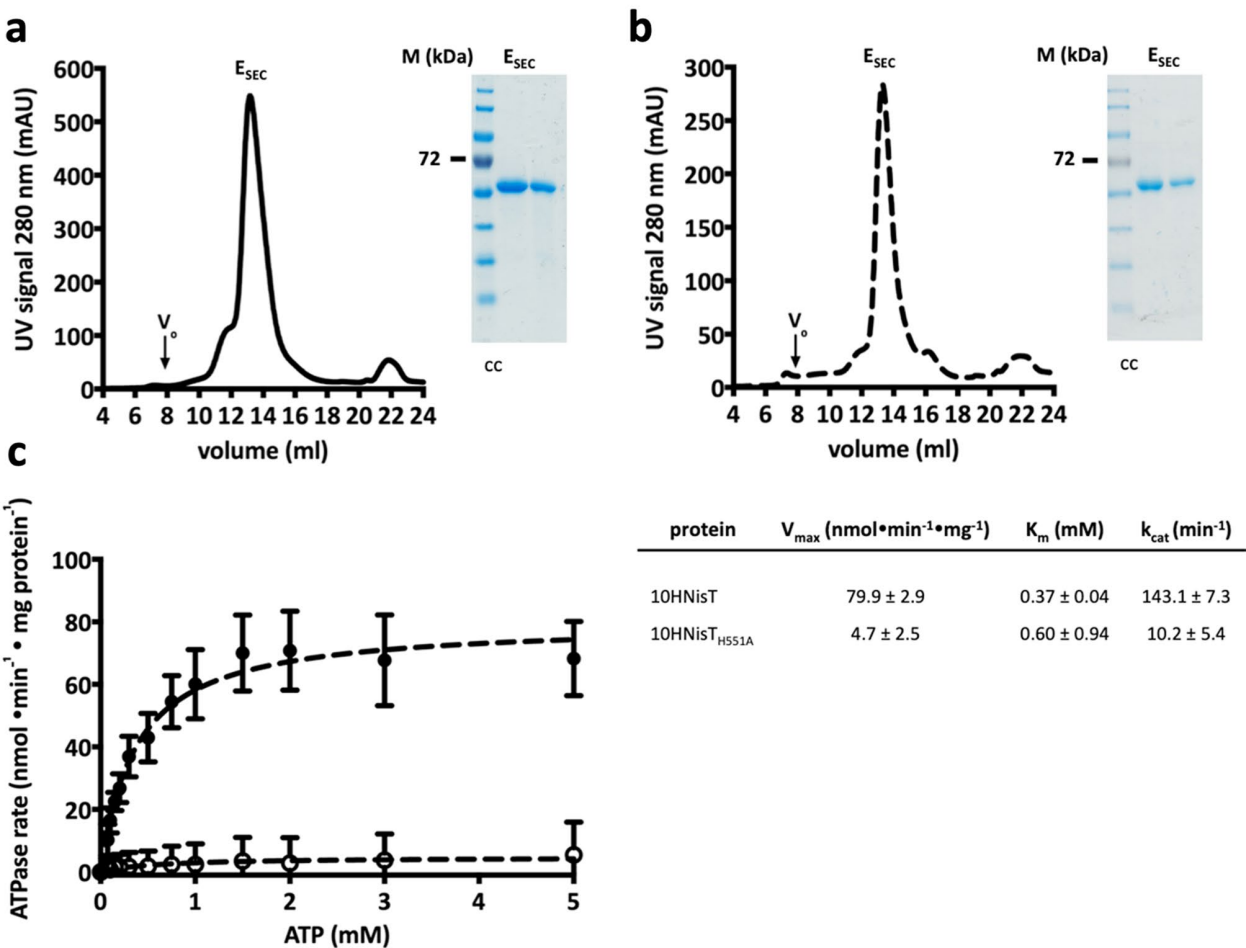
Purification and ATPase activity assay of NisT. (**a**) SEC chromatogram of 10HNisT (WT, black line) displayed a homogeneous peak (E_SEC_) at 13 ml on a Superose 6 10/300 GL column (V_0_: void volume of the column). Inset: A typical colloidal Coomassie (cc) stained SDS-PAGE gel shows a protein band between 55 and 72 kDa marker protein bands (M). (**b**) Purification of the H-loop mutant 10HNisT_H551A_ (HA, dashed line) showed comparable results for SEC profile and SDS-PAGE gel (inset). (**c**) The specific ATPase rate (nmol·min^−1^·mg protein^−1^) of purified WT (black dot) and HA mutant (unfilled circle) was plotted against ATP concentration (mM) to determine kinetic parameters. The ATPase rate was fitted by Michaelis–Menten equation to determine V_max_ (nmol·min^−1^·mg protein^−1^), K_m_ (mM) and k_cat_ (min^-1^). Activity assays were performed from five independent experiments with three replicates and are represented as means ± s.d. (n = 5).

In order to determine the in vitro activity of a lanthipeptide ABC transporter, we purified NisT the exporter of the class I lanthipeptide nisin as a deca-histidine tagged protein variant (10HNisT). 10HNisT was homologously expressed in *L. lactis* NZ9000 and purified (Fig. 3a) after solubilization with the lipid-like surfactant Fos-choline-16 (FC-16, Anatrace) by immobilized metal ion affinity (IMAC) and size-exclusion chromatography (SEC). Similar to other membrane proteins, 10HNisT (72.5 kDa) showed a higher mobility on the SDS-PAGE gel and migrated at approximately 60 kDa. 10HNisT eluted as a homogeneous peak from SEC (Fig. 3a), subsequently the main elution fractions were further concentrated to 50 µM and used for ATPase activity assay.

For the ATPase activity assay, the detergent was exchanged to CYMAL5 (Anatrace) and the ATPase rate was expressed as specific ATPase rate (nmol·min^−1^·mg^−1^). The kinetic parameters of 10HNisT in detergent solution were determined and resulted in V_max_, K_m_ and k_cat_ values for the transporter without its substrate (basal ATPase activity). The concentration of 10HNisT was kept constant (1 µM), whereas the concentration of ATP was varied from 0 to 5 mM and the reaction was stopped after 30 min. The basal ATPase rate of 10HTNisT had a V_max_ value of 79.9 ± 2.9 nmol·min^−1^·mg^−1^, a K_m_ value of 0.37 ± 0.04 mM resulting in a k_cat_ value of 143.1 ± 7.3 min^−1^ (Fig. 3c). As a control the H-loop mutant of 10HNisT (10HNisT_H551A_; HA-mutant) was also purified following the same protocol and used in the ATPase activity assay (Fig. 3b). The ATPase rate of the HA-mutant was reduced by a factor of 17 (V_max_ value 4.7 ± 2.5 nmol·min^−1^·mg^−1^). The K_m_ value increased by a factor of 1.62 (0.60 ± 0.94 mM), whereas the k_cat_ value was 10.2 ± 5.4 min^−1^ (14-fold lower than WT 10HNisT) (Fig. 3c).

### In vitro ATPase activity with NisA variants

To investigate the effect of substrate on the ATPase rate of 10HNisT, we added different NisA variants. First, the NisA peptides in different modification states (uNisA, dNisA and mNisA, respectively) were purified (Figure S4). Additionally, the leader peptide of NisA (NisA_LP_) was used in the ATPase assay to evaluate whether the isolated LP is sufficient for recognition by NisT.

**Figure 4:**
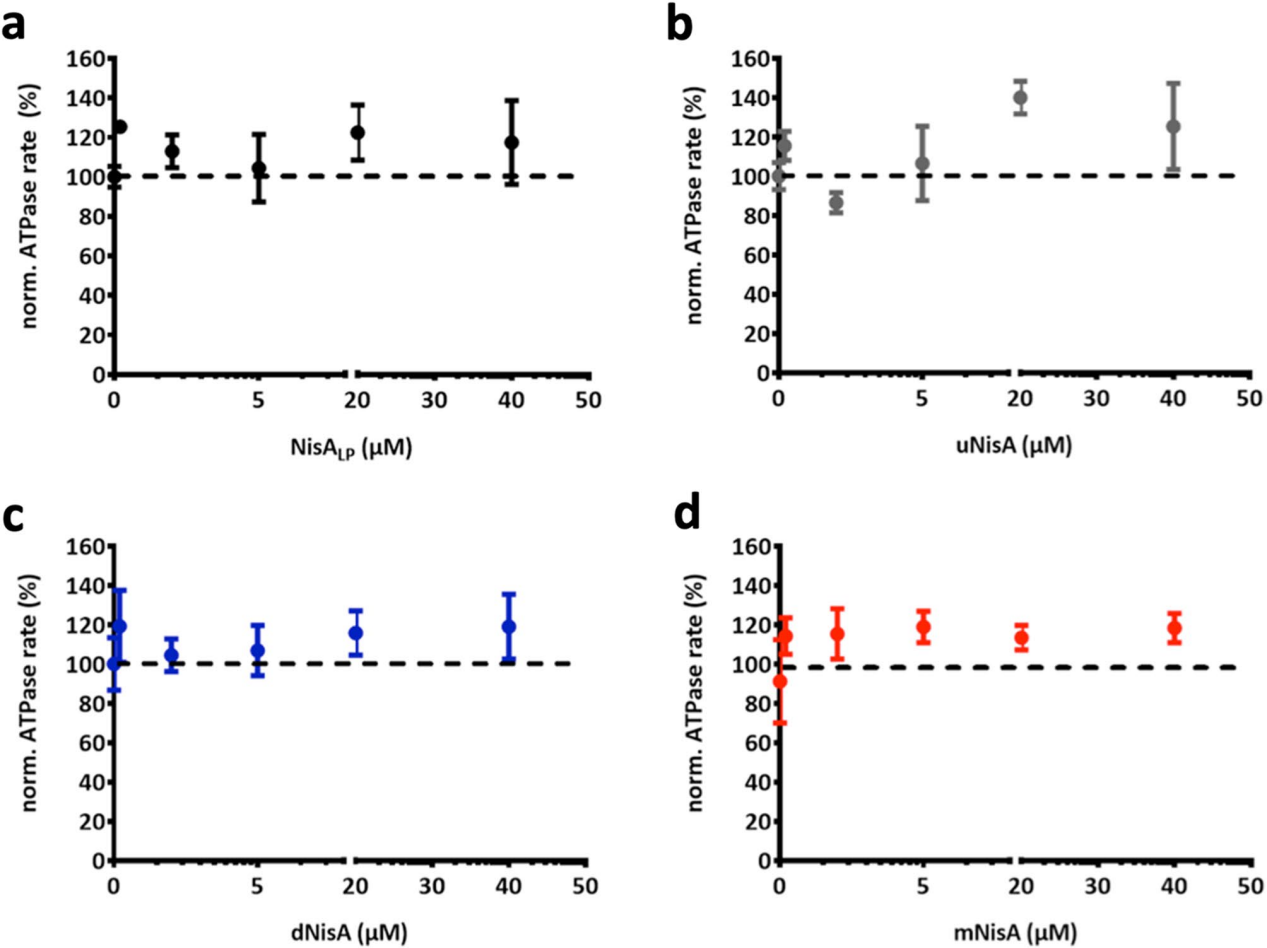
Dependence of NisT ATPase rate on different substrates. The ATPase rate of purified 10HNisT was analyzed in the presence of different substrates. (**a**) The leader peptide of NisA (NisA_LP_, black dots), (**b**) uNisA (grey dots), (**c**) dNisA (blue dots) and (**d**) mNisA (red dots) was used in various concentrations (µM) and the ATPase rate is shown as normalized ATPase rate (%). The basal ATPase rate (from 10HNisT without any substrate; compare Fig. 3) was set as 100% (dashed line) and further values were normalized accordingly. The assays were performed in at least four independent experiments and are represented as means ± s.d. (n = 4). The means were analyzed by a one-way ANOVA and the differences were not significant (p-value: ≥ 0.05). ns: not significant.

For the activity assay the ATP concentration was kept constant at 5 mM, while the substrate concentration was varied from 0 to 40 µM. 10HNisT was pre-incubated with the peptides prior to the activity assay. The basal activity of 10HNisT was set to 100% and the ATPase rate with substrates was expressed as normalized ATPase rate. The ATPase rate of 10HNisT was slightly increased for all peptides. However, a concentration dependent stimulation of the transporter was not observed for all the different tested peptides (Fig. 4a,b) suggesting that the ATPase is not modulated by the NisA variants under the experimental conditions.

### In vitro ATPase activity in presence of NisBC and mNisA

**Figure 5:**
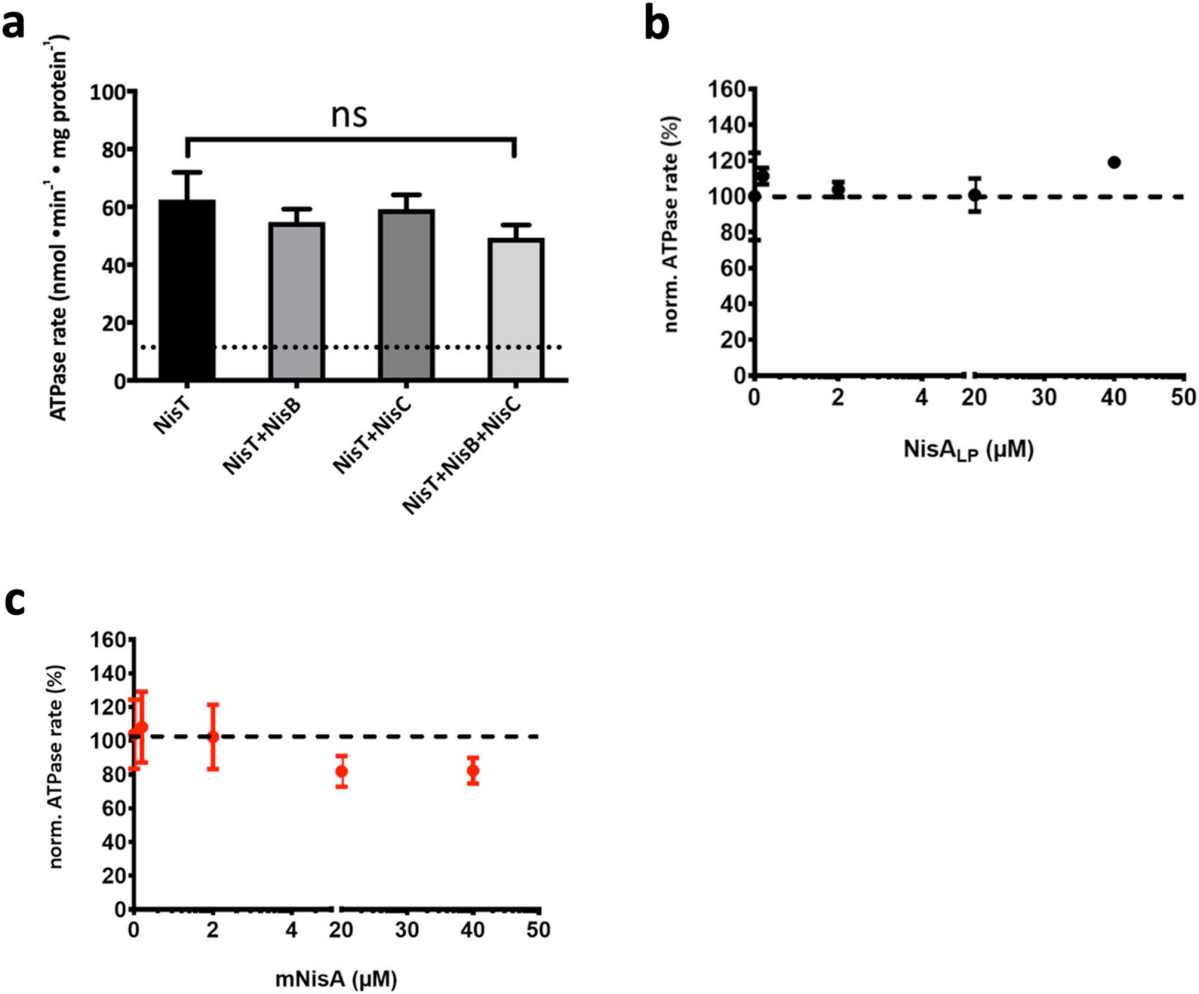
Influence of NisBC on the ATPase rate of NisT. The ATPase rate of purified 10HNisT was analyzed in the presence of the modification enzymes NisB and NisC, respectively. (**a**) ATPase rate of 10HNisT was plotted against variation of 10HNisT with the modification enzymes NisB, NisC and NisBC. It showed the normalized ATPase rate, in which the ATPase rate of 10HNisT was set to 100% (dashed line). (**b**) The substrate NisA_LP_ (black dots) and (**c**) mNisA (red dots) were used in the assay with 10HNisT in presence of NisBC. The normalized ATPase rate was plotted against various concentrations (µM), where the ATPase rate of 10HNisT plus NisBC was set to 100% (dashed line). All assay assays were performed in at least four independent experiments and are represented as means ± s.d. (n = 4). The means were analyzed by a one-way ANOVA and the differences were not significant (p-value: ≥ 0.05).

Previous studies^35^ and our data demonstrated that the secretion of NisA is strongly enhanced by the modification proteins NisB and NisC (see in vivo secretion assay) and therefore the ATPase rate of NisT might also be influenced by these interaction partner. To investigate the effect of NisB and NisC on the ATPase rate of 10HNisT the ATPase activity assay was repeated under the same conditions (ATP concentration constant, various concentration of interaction partners). We observed that the ATPase rate of NisT was independent of the various concentrations of NisB or NisC. Thus, only fixed molar ratio of 10HNisT to the interaction partner was used (NisT:NisB/NisC 1:2; in the case of NisT:NisBC 1:2:2) (Fig. 5a). The basal ATPase rate of NisT was 62.5 ± 9.4 nmol·min^−1^·mg^−1^ and was not changed within the experimental error in the presence of NisB or NisC (54.7 ± 4.5 nmol·min^−1^·mg^−1^, 59.3 ± 4.9 nmol·min^−1^·mg^−1^). If both proteins were used in the assay (NisBC), the ATPase rate of 10HNisT was reduced by a factor of 1.3 (49.3 ± 4.4 nmol·min^−1^·mg^−1^), but the difference was not significant (Fig. 5a).

Next, the ATPase rate of NisT with NisBC was investigated in presence of NisA_LP_ and mNisA, at concentrations ranging from 0 to 40 µM. The ATPase rate with the substrate NisA_LP_ was slightly increased but not in a concentration dependent manner (Fig. 5b), while a decreasing effect on the ATPase rate was observed in presence of mNisA. However, a concentration dependent is not observed and the change is within the experimental error (Fig. 5c).

### Interaction of NisT with NisBC

In 2017 the assembly of the nisin modification complex consisting of NisB_2_C and NisA was published and shed light on the stoichiometry and structure of the complex in solution^41^. Additionally, an influence of the last ring on complex disassembly was determined. The next step would be the interaction with NisT prior to secretion, as a proposed transient multimeric nisin modification/secretion complex^28^, but detailed information about the interaction with the ABC transporter NisT is still missing.

**Figure 6:**
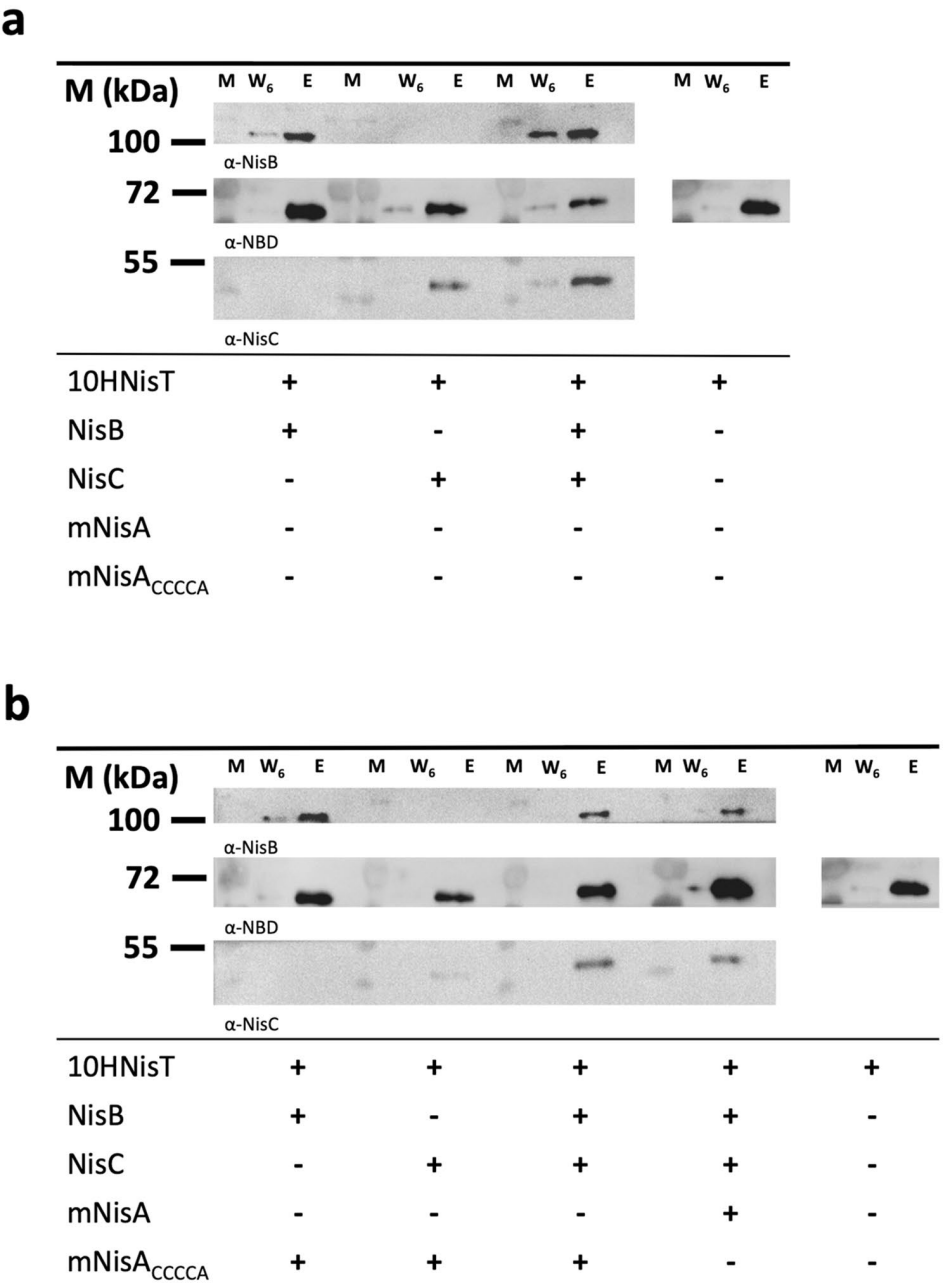
Pull-down assay of NisT with NisB and NisC. The interaction of 10HNisT with NisB and NisC was studied by a pull-down assay. The ABC transporter was immobilized on NTA-magnetic beads, the specific interaction partner were added and incubated. After six washing steps, the last washing step (W_6_) and the EDTA elution fraction (E) were analyzed by Western blot with the specific antibodies (α-NDB, α-NisB or α-NisC). Western blots displayed the eluted bands for NisB, NisT and NisC without substrate (**a**) and with substrates mNisA or mNisA_CCCCA_ (**b**). The pull-down assay was repeated three times and showed similar results. M: marker protein bands; + : protein was used in assay; −: protein was not used in assay.

Therefore, the interaction of NisT with NisB and NisC was investigated by a pull-down assay, in which 10HNisT was immobilized on Ni-NTA-magnetic beads (Qiagen). Initially, the interaction of NisT was tested with 10 µM NisB, NisC or NisBC. Interestingly, in all cases the interaction partner of NisT were observed in the elution fractions. This clearly shows a specific interaction of NisB and NisC with NisT independent of NisA (Fig. 6a). The controls, in which the Ni-NTA-magnetic beads were incubated with NisB and NisC without immobilized 10HNisT, revealed no protein in the elution fractions (Figure S5).

The same set up was used in the presence of the substrate mNisA_CCCCA_, which lacks the last lanthionine-ring (ring E) and stabilizes the maturation complex of NisBC^41^. This substrate showed no additional effect on the interaction of NisB with NisT. However, the interaction of NisT and NisC was affected and the amount of co-eluted NisC was strongly reduced (Fig. 6b). After addition of NisB, the interaction of NisB and NisC with NisT was restored (Fig. 6b). Noteworthy, the addition of mNisA instead of the ring-mutant shows an identical result on complex formation. Unfortunately, the analysis of all elution fractions with an antibody against the LP gave no signals for the substrates mNisA/mNisA_CCCCA_. This might be due to low concentrations of the peptides in the elution fractions.

In summary, this is the first time that beside the interaction of NisT and NisC, an interaction of NisT with NisB was shown. Even the co-elution of NisB and NisC with NisT as a complex in the presence of the substrates mNisA and the ring-mutant mNisA_CCCCA_ was observed. Although no enhancement of the interaction between NisT and NisBC in presence of substrates mNisA/ mNisA_CCCCA_ was detected, NisB seems to have a pivotal role for complex formation.

## Discussion

The mechanism of lanthipeptide modifications was subject to many studies, but still only little is known about the secretion process of class I lanthipeptide ABC transporters (LanT). Only a few in vivo studies investigated the translocation of lanthipeptides nisin, subtilin, Pep5 or epidermin^5,25,42–44^. Amongst these systems, the nisin modification and secretion system (NisBTC) is the best studied one and is commonly employed to secrete nisin variants, lanthipeptides or non-lanthionine containing peptides^5,31,37^. It is commonly established, that nisin is ribosomally synthesized as a precursor peptide NisA which undergoes special post-translational modifications (e.g. Dha, Dhb, Lan and MeLan)^11,45,46^. The PTM of the CP are installed in a coordinated manner by the nisin modification complex NisB_2_C^41,47^.

Not only is the modification is a tightly coupled process, but also the secretion of mNisA by NisT. A proposed channeling mechanism through interaction with NisB/NisC might explain mNisA translocation. Here, mNisA can be detected in the medium within the first minute after induction^35^. We characterized the LanT-type transporter NisT with respect to the secretion process and its specific ATPase activity. To study the mechanism of nisin secretion, we focused on two main topics: (I) In vivo secretion rate of NisA by NisT and (II) in vitro activity of NisT with and without substrate.

In 2008 van den Berg van Saparoea et al*.* conducted a kinetic analysis of nisin production with the strains NZ9700 and NZ9000 transformed with a two plasmid system^35^. They demonstrated distinct contributions of the modification enzymes NisB and NisC with respect to lanthipeptide secretion and proposed that the secretion process of NisT occurred via a channeling mechanism. This hypothesis was supported by further in vivo studies, in which some mechanistic aspects of NisB and NisC modification were investigated^19,47,48^. Although these studies clearly demonstrated the dependence of NisA secretion on the modification enzymes, they did not include a determination of the underlying kinetic parameters. To determine these kinetic parameters, we quantified the amount of secreted peptide via HPLC from different time points of various NZ9000 strains.

The first two kinetic parameters were V_max_ and K_0.5_, which were obtained by an allosteric sigmoidal analysis (Fig. 2). Generally, our results are consistent with the aforementioned studies, in which strain NZ9000BTC had the highest V_max_ and the lowest K_0.5_ value reflecting a high secretion efficiency. The strains, which secreted uNisA (NZ9000T and NZ9000TC), show lower V_max_ and higher K_0.5_ values. Interestingly, we observed in our secretion assay some aberrations with respect to dNisA secretion. The expression of a catalytic-inactive NisC (H331A mutant) in the NisBTC system (strain NZ9000BTC_H331A_) did not restore the secretion level of dNisA to the WT level. This is in contrast to Lubelski et al*.*, where a recovery of the NisA secretion to WT level was observed^19^. The secretion of dNisA by NZ9000BT has a higher V_max_ level and a lower K_0.5_ value. However, one has to consider the time scale of the secretion assays, which might explain this difference. In our assay, the early kinetics of apparent secretion (also see^35^) might pronounce the differences between the strains more clearly as an end-point determination after an overnight secretion. The precise determination of the apparent secretion efficiency revealed a descending order of the NisA secreting strains (NZ9000BTC > NZ9000BT > NZ9000BTC_H331A_ > NZ9000T > NZ9000TC). The secretion efficiency clearly shows, that mNisA is secreted at high rates by strain NZ9000BTC and every aberration of the secretion system reduced the rate at least by a factor of 2.2 (see strain NZ9000BT). The third kinetic parameter was the apparent secretion rate V_S app._ (NisA·NisT^−1^·min^−1^), which we determined for the strains NZ9000BTC and NZ9000NisT, in which the secretion efficiency was the highest for NisBTC system (84.8 ± 21 NisA·NisT^−1^·min^−1^; Fig. 2). This is 9.6-fold higher than with the transporter NisT alone (8.8 ± 1.68 NisA·NisT^−1^·min^−1^).

We observed similar results like other ABC transporter depended secretion system (e.g. HlyA T1SS^49^) that ATP hydrolysis is essential for the secretion process. As the H551A mutant of NisT (strain NZ9000BT_H551A_C) did not secrete mNisA. This mutant enables ATP binding and NBD dimerization but not ATP hydrolysis^50^.

Thus, ATP hydrolysis is clearly important for ABC transporter mediated substrate translocation, we determined the in vitro activity of NisT in terms of ATPase rate without and with substrate. Here, the basal ATPase activity had a value of 79.9 ± 2.9 nmol·min^−1^·mg^−1^ with a K_m_ value of 0.37 ± 0.04 mM, which is in the range of other ABC transporters^51–54^ (Fig. 3). In comparison to NisT, the Lan_C39P_T transporter NukT has a low V_max_ value of 12.6 nmol·min^−1^·mg^−1^, but it is stimulated by mNukA up to max. 500% (at 50 µM substrate). The cleaved substrate stimulates up to 200% (at 25 µM substrate) and the unmodified substrate does not stimulate at all^55^. In the case of NisT, we did not observe a substrate concentration dependent stimulation in the presence of NisA_LP_, uNisA, dNisA and mNisA (Fig. 4). Next, we extended the ATPase activity by addition of NisB and NisC to simulate NisBTC, in which the secretion of NisA is the most efficient (Fig. 5). The addition of Nis_LP_ had no effect on the ATPase rate. However, the addition of mNisA revealed an inhibiting effect on the ATPase rate with increasing substrate concentration, although it was within the experimental error. Interestingly, a similar behavior was observed for PCAT1^52^. Another study on an ABC transporter homologue to PrtD from *Aquifex aeolicus* also noticed an inhibitory effect on ATPase activity after substrate addition^56^. The open question is now, if NisT uses an equivalent mechanism, in which the interaction of the modification/ secretion complex inhibits the ATPase rate prior to translocation.

In 1996 a multimeric enzyme complex of NisBTC was proposed, but the isolation of such a complex was not successful^28^. Therefore, we choose to study the specific interaction of NisT with NisB and NisC via a pull-down assay. Such a pull-down assay was performed with His-tagged NisA, where NisB and NisC were co-eluted from cytoplasmic fraction^47,57^. In our study, we expanded this set up and used purified NisT, NisB, NisC and NisA (Fig. 6). We observed a specific interaction of NisT with NisC, which is in line with previously observed interaction of NisT with NisC via co-immunoprecipitation and yeast two-hybrid assay^28^. Another specific interaction of the modification enzyme with the ABC transporter was shown for NukM and NukT. Here, the C-terminal domain of LanM (LanC-like domain; amino acids 480–917) interacts with TMD and NBD of NukT, but not with the C39 peptidase domain^30^. Besides the interaction of NisT with NisC, we also noticed an interaction of NisT with NisB, which was not observed in the above-mentioned study, but for SpaT and SpaB in a similar experiment^29^. Since secretion of dNisA by NZ9000BT was observed^5^, an interaction of NisT and NisB was also proposed. Furthermore, we observed for the first time the co-elution of NisBC with NisT in the proposed lanthionine synthetase complex. Remarkably, the co-elution of transporter with the modification enzymes is not increased by addition of the substrates mNisA or mNisA_CCCCA_. Similar amounts of the enzymes were co-eluted and we conclude that the interaction of NisT with NisB and NisC is independent of substrate. One exception was the addition of mNisA_CCCCA_ to NisT/NisC, in which the amount of co-elute NisC was reduced. Only the addition of NisB to the sample increased the co-elution of NisC. It is now commonly accepted that NisB represents the main component of the NisBTC modification/secretion complex^19,35^ and based on our data, NisB stabilizes the NisBTC complex.

In summary, we have determined the kinetic parameters for the in vivo secretion of nisin peptides by the modification and secretion complex NisBTC. We demonstrated that alterations in the NisBTC system lead to impaired secretion of NisA or even to no secretion, when an ATP hydrolysis deficient mutant of NisT was used. For an efficient secretion by NisT the modification enzymes NisB and NisC are prerequisite and their interaction with NisT enhances the secretion process directly by the proposed channeling mechanism^35^. Interestingly, it is the function not the mere presence of NisC that is required, as the secretion of NisBTC with the catalytically inactive mutant of NisC did not restore the secretion. Additionally, the in vitro activity data of NisT demonstrate that the lanthipeptide exporter is not stimulated by the interaction partner and substrate, respectively. The observed complex formation of NisB and NisC with NisT hint to the proposed lanthionine synthetase complex, in which NisT under goes a transition to a transport competent exporter in presence of only NisB, NisC and the substrate mNisA.

## Methods

### Bacterial strains and growth conditions

Strains of *Escherichia coli* and *Lactococcus lactis* and plasmids used in this study are listed in Table S1. The strains *E. coli* DH5α or BL21 were grown in LB medium at 37 °C under aerobic conditions with appropriate antibiotics (30 μg/ml kanamycin or 100 μg/ml ampicillin). The transformation of *E. coli* strains was performed following standard procedures.

The strain *L. lactis* NZ9000 (and its variants) was grown in M17^58^ or minimal medium (MM)^31,59^ at 30 °C under semi aerobic conditions supplemented with 0.5% glucose (GM17/ GMM) and appropriate antibiotics (erythromycin or/and chloramphenicol at a final concentration of 5 μg/ml). To MM a vitamin mix (100 × stock solution, 1 × final) was added^31^.

For transformation of *L. lactis* NZ9000 with the expression plasmids a standard procedure for preparation of competent cells and electroporation was used^60^.

### Cloning of nisT and nisT variants

A nucleotide sequence for a MCS with 10H nucleotide sequence (5′–3′: CAAAATAAATTATAAGGAGGCACTCAATGCATCATCATCATCATCATCATCATCATCATGATTATGATATTCCGACCACCGAAAACCTGTATTTTCAGGGCCCATGGCATATGCATATGTCTAGACACCACCACCACCACCACTGAGATCCG) was ordered as a codon-optimized, synthetic gene fragment from Life Technologies to insert it into the pNZ-SV plasmid^61^. The synthetic gene fragment were amplified by Phusion DNA polymerase (NEB) with the primer pair 10Hfor and 10Hrev (Table S4) for Gibson assembly. The plasmid pNZ-SV was amplified by Phusion DNA polymerase (NEB) with the primer pair infupNZ-SVfor and infupNZ-SVrev (Table S4) to linearize the vector. The gene fragment and the vector pNZ-SV were employed in the Gibson assembly by following the manufactures instructions (NEB). The Gibson assembly reactions were transformed into *E. coli* DH5α. The sequence of the construct pNZ-SV10H (Table S5) was verified by DNA sequencing (Microsynth Seqlab).

The *nisT* gene (accession number: Q03203) was amplified using genomic DNA from *L. lactis* NZ97000^8^ as a template. Phusion DNA polymerase (NEB) with the primer pair infunisTfor and infunisTrev (Table S4) was used to create overhang sequences for Gibson assembly. The plasmid pNZ-SV10H was amplified by Phusion DNA polymerase (NEB) with the primer pair linpNZ-SVfor and linpNZ-SVrev (Table S4) to linearize the vector. Subsequently, the gene and the linearized vector pNZ-SV10H were employed in the Gibson assembly and the reactions were transformed into *E. coli* DH5α. The sequence of the construct pNZ-SV10HnisT (Table S5) was verified by DNA sequencing (Microsynth Seqlab).

To generate the plasmid pIL-SVnisT the *nisT* gene from pNZ-SV10HnisT was amplified by Phusion DNA polymerase (NEB) with the primer pair infupIL-SVfor and infupIL-SVrev (Table S4) to create overhanging sequences. The plasmid pIL-SV^62^ was linearized by Phusion DNA polymerase (NEB) with the primer pair termpNZfor and pnisArev (Table S4). The gene with overhang sequences and the vector was employed in Gibson assembly. The Gibson assembly reactions were transformed into *E. coli* DH5α. Additionally, the 10H nucleotide sequence was deleted by Phusion DNA polymerase (NEB) with the primer pair 10Hfor and infupNZ-SVrev (Table S4). The sequence of the construct pIL-SVnisT (Table S5) was verified by DNA sequencing (Microsynth Seqlab).

To generate the *nisT*_H551A_ mutant, a polymerase chain reaction using Pfu DNA polymerase (Thermo Fischer Scientific) or Pfu DNA polymerase (Promega), the template pNZ-SV10HnisT or pIL-SVnisT and the primer pair nisT_H551A_for and nisT_H551A_rev (Table S4) was performed according to standard procedures. The sequence of the constructs (Table S5) was verified by DNA sequencing (Microsynth Seqlab).

The plasmid pNZ-SVnisTNBD_H348_ was obtained by the deletion of the TMD sequence (1–347) from the plasmid pNV-SV10HnisT. The plasmid was amplified with Phusion DNA polymerase (NEB) with the primer pair ΔnisT_TMD_for and ΔnisT_TMD_rev (Table S4). The linear vector was ligated with T4-ligase (NEB) and transformed into *E. coli* DH5α. The sequence of the construct pNZ-SVnisTNBD_H348_ (Table S5) was verified by DNA sequencing (Microsynth Seqlab).

### Cloning of nisBTC and nisBTC variants

The plasmid pIL-SVnisBTC was generated from pIL-SV and pIL3BTC^31^. The plasmid pIL3BTC was digested with the restriction enzymes NotI (NEB) and BstXI (NEB) to receive a fragment BTC containing the genes *nisB*, *nisT* and *nisC*. Next, pIL-SV^62^ was also digested with NotI and BstXI (pIL-SV**). The fragment BTC and pIL-SV** were ligated with T4-ligase (NEB) and transformed into *E. coli* DH5α. The sequence of the construct pIL-SVnisBTC (Table S5) was verified by DNA sequencing (Microsynth Seqlab).

By using Phusion DNA polymerase (NEB) with the appropriate primer pairs (Table S4) the gene deletions of *nisB*, *nisC* or *nisT* were performed to generate pIL-SVnisBTC derivatives. Subsequently, the linearized vectors were ligated with T4-ligase (NEB) and transformed into *E. coli* DH5α. The sequence of the constructs (Table S5) were verified by DNA sequencing (Microsynth Seqlab).

To generate *nisT*_H551A_ and nisC_H331A_ mutants, a polymerase chain reaction using PfuUltra II Fusion DNA polymerase (Agilent Technologies), the template pIL-SVnisBTC and the appropriate pair of oligonucleotides (Table S4) was performed according to standard procedures. The sequence of the new constructs (Table S5) were verified by DNA sequencing (Microsynth Seqlab).

### In vivo secretion assay: expression and secretion of NisA

Strain *L. lactis* NZ9000 harboring the plasmids pIL-SVnisBTC and pNZ-SVnisA hereupon termed NZ9000BTC (Table S1) was used to investigate the in vivo secretion activity of the nisin modification and secretion system (NisBTC). The use of pIL-SVnisBTC derivatives and pNZ-SVnisA for transformation into *L. lactis* NZ9000 led to strains described in detail in Table S1. For each secretion experiment new transformants were prepared and used to inoculate GM17 (Erm + Cm) with one colony. The overnight culture was centrifuged at 4,000×*g* for 20 min and cells were resuspended in GMM. Subsequently, 0.5 l GMM (Erm + Cm) were inoculated to OD_600_ of 0.3 and incubated at 30 °C. After 60–90 min the culture (OD_600_ of 0.4–0.5) was induced with 10 ng/ml nisin (powder from Sigma-Aldrich dissolved in 50 mM lactic acid). A 50 ml sample before induction (0 h) and every other hour (1–6 h) was taken. For each sample the cell were harvested by centrifugation at 4,000×*g* for 20 min. Subsequently, the cells were resuspended in R-buffer (50 mM Na-Phosphate buffer, pH 8, 100 mM KCl, 20% glycerol) to an OD_600_ of 200, flash frozen in liquid nitrogen (N_2_) and stored at − 80 °C until further use. The supernatant was additionally centrifuged at 17,000×*g* for 20 min at 8 °C. Supernatants were kept on ice before the RP-HPLC analysis. Furthermore, 2 ml or 10 ml of the supernatant were precipitated by 1/10 volume (10%) TCA. TCA samples were incubated at 8 °C overnight. The TCA-precipitated peptide was centrifuged at 17,000×*g* for 20 min at 8 °C and consecutively washed three-times with ice-cold acetone. The pellets were vacuum-dried and resuspended in 60 µl per OD_600_ 1 of 1 × SDS-PAGE loading dye containing 5 mM β-mercaptoethanol (β-ME). These resuspended TCA-pellets were analyzed by Tricine-SDS-PAGE and Western blot.

### In vivo secretion assay: analysis of cell pellets

The resuspended cell pellets were thawed on ice and 1/3 (w/v) glass beads (0.3 mm diameter) were added. Cells were disrupted on a vortex-shaker (Disrutor Genie, Scientific Industries). A cycle of 2 min disruption and 1 min incubation on ice was repeated five times. A low spin step at 17,000×g for 30 min at 8 °C and subsequently a high spin step at 100,000×g for 120 min at 8 °C was performed. The supernatant of the latter centrifugation step represents the cytoplasmic fraction and the pellet corresponds to the membrane fraction. The SDS-PAGE samples of cytoplasmic and membrane fractions were prepared by adding 4 × SDS-PAGE loading dye containing 5 mM β-ME and used for SDS-PAGE as well as Western blot analysis.

### In vivo secretion assay: analysis of culture supernatant

The culture supernatants containing NisA variants were analyzed by RP-HPLC (Agilent Technologies 1260 Infinity II). A LiChrospher WP 300 RP-18 end-capped column and an acetonitrile/water solvent system were used as described previously^7^. In the case of modified NisA 300 µl and for all other variants (dehydrated/unmodified) 500 µl sample were injected. Prior to the gradient (20–50% acetonitrile) a washing step of 20% acetonitrile was used to remove most of the casein peptides. The peptide amount or NisA in the supernatant was determined using the peak area integration analyzed with the Agilent Lab Advisor software. In the case of the unmodified peptide (uNisA) a retention time from 29 to 33 min, for dehydrated peptide (dNisA) from 31 to 38 min and for modified peptide (mNisA) from 33 to 38 min was used for peak integration peak. A calibration with known amounts of nisin or insulin chain B was used to obtain a linear regression line. Unknown amounts in the in vivo secretion assay samples were calculated as nmol or µM based on this linear regression line.

### In vivo secretion assay: determination of kinetic parameter

The amount of secreted NisA of the different *L. lactis* NZ9000 strains were plotted against time and fitted using an allosteric sigmoidal fit (1). Note that y is the amount of secreted peptide (nmol), V_max_ the maximal secreted amount, x is time (min), K_0.5_ the time point at which 50% of V_max_ is present and h is the Hill slope indicating cooperativity. The analysis was performed using Prism 7.0c (GraphPad).

1 $$y={V}_{max}\frac{{X}^{h}}{{{K}_{0.5}}^{h}+{X}^{h}}$$

The apparent secretion rate (V_s app_) was determined by plotting the amount of NisA and NisT against time (min). The values were fitted using a linear regression (2). Note that y is the amount of NisA molecules per NisT molecules (NisA·NisT^−1^), m is the slope V_s app_ (NisA·NisT^−1^·min^−1^), x is the time (min), and b the y-axis interception. The analysis was performed using Prism 7.0c (GraphPad).

2 $$y=mx+b$$

### Expression and purification of NisT

*L. lactis* NZ9000 strain was transformed with pNZ-SV10HnisT and placed on SGM17 (0.5 M sucrose, 0.5% glucose, 22 g/l agarose in M17 medium) agar plates containing 5 µg/ml erythromycin. A GM17 (Erm) overnight culture was inoculated with one colony and incubated at 30 °C. A GM17 (Erm) main culture was inoculated to an OD_600_ of 0.1 with the overnight culture. After 3 h incubation, expression was induced by adding 10 ng/ml nisin (powder from Sigma-Aldrich dissolved in 50 mM lactic acid) and further grown for additional 3 h. Cells were harvested by centrifugation at 4,000×g for 20 min at 8 °C and resuspended in R-buffer (50 mM Na-Phosphate buffer, pH 8, 100 mM KCl, 20% glycerol) to an OD_600_ of 200. To the resuspended cells 10 mg/ml lysozyme was added and incubated at 30 °C for 30 min. Prior to cell disruption, cells were incubated on ice for 15 min. The cell suspension was passed through a homogenizer (M-110P, Microfluidics System) at 1.5 kbar at least four times. The homogenized cell suspension was centrifuged at 12,000×g for 30 min at 8 °C. Subsequently, the supernatant was centrifuged at 100,000×g for 120 min at 4 °C to collect the membrane fraction. Membranes were resuspended with R-buffer containing 10 mM imidazole and 0.5 mM AEBSF. The total membrane protein concentration was measured by BCA assay (Thermo Fischer Scientific) and the concentration was adjusted to 5–7.5 mg/ml. Membranes were solubilized with 1% (w/v) of the lipid-like detergents FC-16 (Anatrace) for 1 h at 8 °C. Insoluble material was removed by centrifugation at 100,000×g for 30 min at 4 °C. The supernatant was applied to a 5 ml IMAC (Immobilized Metal-Ion-Affinity Chromatography) HiTrap Chelating column (GE Healthcare) preloaded with 100 mM zinc sulphate and equilibrated with low IMAC1 buffer (50 mM Na-Phosphate buffer, pH 8, 100 mM KCl, 20% glycerol and 10 mM imidazole) containing 0.5 mM AEBSF and 0.005% FC-16. Consecutively, non-bound protein was washed and the buffer was exchanged to low IMAC2 buffer (50 mM Tris–HCl pH 8, 100 mM KCl, 10% glycerol, 10 mM imidazole, 0.5 mM AEBSF and 0.005% FC-16). After an additional washing step with 30% high IMAC buffer (50 mM Tris–HCl pH 8, 100 mM KCl, 10% glycerol, 150 mM histidine, 0.5 mM AEBSF and 0.005% FC-16), 10HNisT was eluted with 100% high IMAC buffer. The elution fractions containing NisT were pooled, 10 mM DTT was added and further concentrated with a Vivaspin20 100 kDa molecular weight cut off (MWCO) centrifugal concentrator (Sartorius AG). Next, a size exclusion chromatography (SEC) was performed, where the concentrated protein sample was applied onto a Superose 6 10/300 GL column (GE Healthcare) equilibrated with SEC buffer (25 mM Tris–HCl pH 8, 50 mM KCl, 10% glycerol, 0.5 mM AEBSF, 2 mM DTT and 0.0015% FC-16). The main peak fractions were analyzed via SDS-PAGE and further concentrated via a Vivaspin6 100 kDa MWCO centrifugal concentrator (Sartorius AG) until a concentration of 50 µM was reached. The protein concentration was determined by NanoDrop spectrophotometer (Thermo Fischer Scientific) using a molar extinction coefficient of 86,180 M^−1^ cm^−1^ and the molecular mass of 72.6 kDa. Aliquots of 50 µM 10HNisT were flash frozen in liquid N_2_ and stored at − 80 °C until further use. The NisT variants 10HNisT_H551A_ was expressed following the same protocol.

### In vitro ATPase activity assay

The ATPase activity of NisT was determined with the malachite green assay as described previously with experimental alterations^63^. In this assay the release of inorganic orthophosphate after ATP hydrolysis was colorimetrically quantified based on a Na_2_HPO_4_ standard curve.

All reactions were performed at 30 °C in a total volume of 30 μl in activity assay buffer containing 0.4% CYMAL5 and 10 mM MgCl_2_.

In each reaction ~ 2 μg of detergent-solubilized and purified NisT was used and the reaction was started by adding ATP (0–5 mM). The background of the reaction was a sample without MgCl_2_. After 30 min the reaction was stopped by transferring 25 μl of each reaction into a 96-well plate containing 175 μl stop-solution (20 mM sulphuric acid). Consecutively, 50 μl of a staining solution (0.096% (w/v) malachite green, 1.48% (w/v) ammonium heptamolybdate and 0.173% (w/v) Tween-20 in 2.36 M sulphuric acid) was added. After 10 min the amount of free inorganic orthophosphate was quantified by measuring the absorption at 595 nm using an iMark microplate reader (Bio-Rad).

The specific ATPase activity of NisT was plotted against ATP concentrations and fitted using the Michaelis–Menten Eq. (3). Note that y is the reaction velocity, V_max_ the maximal reaction velocity, x is the substrate concentration and K_m_ the Michaelis–Menten constant. The analysis was performed using Prism 7.0c (GraphPad).

3 $$y={V}_{max}\frac{X}{{K}_{m}+X}$$

For the reactions with substrates (NisA variants) or interaction partner (NisB and NisC) NisT was pre-incubated at 30 °C for 10 min before ATP was added to start the reaction. All reactions were performed at 30 °C in a total volume of 30 μl in activity assay buffer containing 0.4% CYMAL5, 400 mM glutamate and 10 mM MgCl_2_. In each reaction ~ 2 μg of detergent-solubilized and purified NisT was used and the reaction was started by adding 5 mM ATP and stopped after 15 min following the procedure described above. In this reaction the concentration of the different substrates (0–40 µM) and/or interaction partner was varied and the ATPase activity was normalized to the specific ATPase activity of NisT without substrate/interaction partner. In these cases, the background was subtracted prior to normalization.

### In vitro pull-down assay

The immobilization of 10HNisT to Ni-NTA magnetic beads (Quiagen) was performed as described in the manufacturer’s manual. In brief, ~ 15 µg 10HNisT was incubated with Ni-NTA magnetic beads for 30 min at 30 °C. Excess of protein was removed by three washing steps with activity assay buffer containing 0.4% CYMAL5 and 400 mM glutamate. 10 µM of the interaction partners NisC and NisB were incubated in 1:1 molar ratio (but > 10 × molar excess to NisT) separately with or without mNisA/ mNisA_CCCCA_ (20 × molar excess to NisT) in activity assay buffer containing 0.4% CYMAL5, 400 mM glutamate and 5 mM MgATP for 15 min on ice. Next, interaction partner were added to 10HNisT immobilized to Ni-NTA magnetic beads and incubated for 1 h at 30 °C. Positive control (only 10HNisT) and negative control (NisB, NisC samples were prepared by incubating the proteins with Ni-NTA magnetic beads separately. After binding the Ni-NTA magnetic beads were washed six times with activity assay buffer. Finally, 10HNisT was eluted by adding activity assay buffer containing 50 mM EDTA. The SDS-PAGE samples of pull-down assay fractions were prepared by adding 4 × SDS-PAGE loading dye containing 5 mM β-ME and used for Western blot analysis.

## Supplementary information

Supplementary Information

## Abbreviations

ABC transporter: ATP binding cassette transporter
CP: Core peptide
Dha: Didehydroalanine
Dhb: Didehydrobutyrine
Lan: Lanthionine
LP: Leader peptide
MeLan: Methyl-lanthionine
MS: Mass spectrometry
NBD: Nucleotide-binding domain
NisA: Precursor peptide of nisin
NisB: Dehydratase of nisin precursor peptide
NisC: Cyclase of nisin precursor peptide
NisT: Transporter of nisin precursor peptide
PTM: Post-translational modificationn
RP-HPLC: Reverse-phase HPLC
SEC: Size-exclusion chromatography
