# Supplementary material for: Impact of the nisin modification machinery on the transport kinetics of NisT
Source: Sci Rep. 2020 Jul 23;10:12295. doi: 10.1038/s41598-020-69225-2 (PMC7378552; doi:10.1038/s41598-020-69225-2)
Supplement: Supplementary file 1 — Supplementary Information [file 41598_2020_69225_MOESM1_ESM.docx]

**Supplemental information**

**Impact of the nisin modification machinery on the transport kinetics of NisT**

Marcel Lagedroste^1^, Jens Reiners^1^, Sander H.J. Smits^1^ and Lutz Schmitt^1^*

^1^:Institute of Biochemistry, Heinrich Heine University Düsseldorf

*: To whom correspondence should be addressed

Lutz Schmitt

Institute of Biochemistry

Heinrich Heine University Düsseldorf

Universitätsstr. 1

40225 Düsseldorf, Germany

Phone:	+49(0)211-81-10773

Fax:	+49(0)211-81-15310

Email:	[lutz.schmitt@hhu.de](mailto:lutz.schmitt@hhu.de)

**Methods**

*Chemicals and Antibodies*

Fos-choline 16 (FC-16) and CYMAL5 (C5) were obtained from Anatrace. Lyophilized nisin powder (2.5% nisin content) and insulin chain B were obtained from Sigma-Aldrich. The leader peptide of NisA was synthesized and obtained from JPT peptide technologies. Antibodies for NisT_NBD_, NisC and NisA_LP_ peptide were purchased from Davids Biotechnology (Germany) as polyclonal antibodies. NisB antibody was kindly provided by Dr. G. Moll (Lanthio Pharma; Groningen, Netherlands). All standard chemicals were purchased from Sigma-Aldrich or VWR.

*Expression and purification of NisT_NBD_*

*L. lactis* NZ9000 was transformed with pNZ-SVnisTNBD_H348_ and placed on SMGG17 agar plates containing 5 µg/ml erythromycin. A GM17 (Erm) overnight culture was inoculated with one colony and incubated at 30°C. A GM17 (Erm) main culture was inoculated to an OD_600_ of 0.1 with the overnight culture. After 90 min incubation the culture was incubated on ice. Then expression was induced by adding 10 ng/ml nisin (powder from Sigma-Aldrich dissolved in 50 mM lactic acid) and further grown for additional 3 h at 20 °C. Cells were harvested by centrifugation at 4000 xg for 20 min at 8°C and resuspended in R-buffer (50 mM Na-Phosphate buffer, pH 8, 100 mM KCl, 20% glycerol) to an OD_600_ of 200. Then 10 mg/ml lysozyme was added and incubated at 30°C for 30 min. Prior to cell disruption, cells were incubated on ice for 15 min. Then the cell suspension was passed through a homogenizer (M-110P, Microfluidics System) at 1.5 kbar at least four times. The homogenized cell suspension was centrifuged at 12,000 xg for 30 min at 8°C. Subsequently, the supernatant was centrifuged at 100,000 xg for 120 min at 4°C. The supernatant was applied to a 5ml IMAC HiTrap Chelating column (GE Healthcare) preloaded with 100 mM zinc sulphate and equilibrated with low IMAC1 buffer (50 mM Na-Phosphate buffer, pH 8, 100 mM KCl, 20% glycerol and 10 mM imidazole). Consecutively, non-bound protein was washed by a 30% step with high IMAC buffer (50 mM Na-Phosphate buffer, pH 8, 100 mM KCl, 20% glycerol, 500 mM imidazole). The protein 10HNisT_NBDH348_ was eluted by a gradient of 30 to 100% high IMAC buffer. The elution fractions containing NisT_NBDH348_ (T_NBD_) were pooled, 10 mM DTT was added and further concentrated with an Amicon 10 kDa MWCO centrifugal concentrator (Millipore). Next, the concentrated protein sample was applied onto a HiLoad Superdex 200 16/60 prep grade column (GE Healthcare) equilibrated with SEC buffer (25 mM CAPS pH 10, 20% glycerol, 2 mM DTT). The main peak fractions are analyzed via SDS-PAGE and further concentrated with an Amicon 10 kDa MWCO centrifugal concentrator (Millipore). The protein concentration was determined by NanoDrop spectrophotometer (Thermo Fischer Scientific) using a molar extinction coefficient of 45,840 M^-1^•cm^-1^ and the molecular mass of 32.2 kDa. Aliquots of 30 µM T_NBD_ were flash frozen in liquid N_2_ and stored at −80 °C until further use.

*Expression and purification of nisin modification enzymes NisB and NisC*

NisB and NisC were expressed and purified as described previously ^1-3^. Aliquots of concentrated proteins (90 µM (NisB) or 110 µM (NisC)) were flash frozen in liquid nitrogen and stored at −80 °C until further use. For the *in vitro* ATPase activity assay and pull-down assay the buffer of the proteins was exchanged via a PD SpinTrap G-25 spin columns (GE Healthcare) to activity assay buffer (25mM Tris-HCl pH 7.5, 50 mM KCl) containing 400 mM glutamate and 0.4% CYMAL5 (3 x cmc). Proteins were stored on ice and directly used for the *in vitro* assays. The protein concentration was determined by NanoDrop spectrophotometer (Thermo Fischer Scientific) by using the theoretical molar extinction coefficient and the molecular mass of the proteins.

*Expression and purification of NisA variants*

All NisA variants used in this study were expressed and purified as described previously with modifications ^1,4^. Briefly, NisA variants were purified after 5 h expression in MM from 2 l (0.5 l in the case of the secretion experiments) culture supernatant via cation-exchange chromatography (cIEX). The cell-free supernatant was diluted 1:1 with LA buffer (50 mM lactic acid, pH 3) and applied to a 5 ml HiTrap SP Sepharose column (GE Healthcare). The column was washed by applying a pH gradient from 100 % LA buffer to 100% H buffer (50 mM HEPES-NaOH, pH 7). Finally, the peptides were eluted with H buffer containing 1 M NaCl. The fractions containing the peptides were pooled and concentrated with an Amicon (Millipore) centrifugal concentrator. First, the flow through (FT) fraction of a 30 kDa molecular weight cut off (MWCO) concentrator was collect. Then a 3 kDa MWCO filter was used to concentrate the peptides of the FT fraction. Aliquots of concentrated peptides were flash frozen in liquid N_2_ and stored at −80 °C until further use. For concentration determination the NisA variants were analyzed by RP-HPLC (Agilent Technologies 1260 Infinity II) with a LiChrospher WP 300 RP-18 end-capped column and an acetonitrile/water solvent system as described previously ^2^.

*SDS-PAGE and immunoblotting for protein/ peptide analysis*

In general the sodium dodecyl sulfate−polyacrylamide gel electrophoresis (SDS-PAGE) experiments were performed using standard procedures ^5^. In the SDS-PAGE gels the acrylamide portion was 10% to have a separation range from 30 to 120 kDa for the proteins NisC (~48 kDa), NisT (~69 kDa) and NisB (~117 kDa).

All peptides (NisA variants) from secretion experiments or from cIEX purification were analyzed by Tricine-SDS−PAGE ^6^. For Tricine-SDS-PAGE gels (12%) a Mini-Protean system (Bio-Rad) was used. Tricine-SDS−PAGE and SDS−PAGE gels were stained with colloidal Coomassie (cc) ^7^.

All immunoblotting experiments were conducted following standard procedures. For the quantification of NisT in the membrane fractions (*in vivo* secretion assay) various amounts of a T_NBD_ standard (stock solution 12.5 µg/ml) was added to create a calibration curve. The band intensities on the Western blots were processed and determined by ImageJ ^8^. Then the amount of NisT was determined as pmol protein of the different membrane fractions of the time points 2-6 h.

*Mass spectrometry analysis*

NisA variants were either desalted via ZipTip (C18 resin) purification accordingly to the manufacture manual (Merk-Millipore) or by RP-HPLC and vacuum dried.

For the MALDI-TOF-MS analysis the vacuum dried pellet was dissolved in 50% acetonitrile solution containing 0.1% TFA and analyzed as described elsewhere ^9^.

**Results** (supporting results section of main manuscript)

***In vivo* secretion assay**

The supernatants of the *in vivo* secretion assay were analyzed by RP-HPLC. The secreted peptides were separated by an acetonitrile/water gradient after a 20% washing step on a C-18 RP-HPLC column. The eluted peptides were monitored via UV signal at 205 nm and collected fractions of 1 ml were collected and used for MALDI-TOF-MS analysis. The MS analysis confirmed the correct masses for NisA peptides (uNisA, dNisA or mNisA) in fractions 30-36 min of the chromatogram (Figure S1). The modified peptide mNisA with eight (-Met: 5689 Da) or seven (-Met: 5707 Da) dehydrations from strain NZ9000BTC was detected in fractions 34-36 min (Figure S1A, Table S2). Furthermore, the modified peptides (dNisA) from strains NZ9000BTC_H331A_ and NZ9000BT with eight dehydrations (-Met: 5689 Da) were found in fractions 33-36 min (Figure S1B/D, Table S2). In the case of the peptide from strains NZ9000T and NZ9000TC the retention time was shifted and the unmodified peptide was eluted in fractions 30-32 min (Figure S1E/F). The corresponding molecular mass for unmodified NisA (+Met: 5951 Da; -Met: 5834 Da) was verified (Table S2).

**Figure S1: RP-HPLC chromatograms and MALDI-TOF-MS spectra of *in vivo* secretion assay.**

The supernatants of NisA secreting *L. lactis* NZ9000 strains were employed for an RP-HPLC analysis. The NisA variants (mNisA, dNisA and uNisA) were separated from other peptides in the supernatant by an acetonitrile/water gradient on a C-18 RP-HPLC column (left panel). The elution fractions (dashed square; middle panel) were further analyzed by MALDI-TOF-MS (right panel) to verify the correct mass. Integration of the corresponding peaks enables the determination of peptide amounts (nmol). Supernatant analysis of *L. lactis* strains (**A**) NZ9000BTC, (**B**) NZ9000BTC_H331A_, (**C**) NZ9000BT_H551A_C, (**D**) NZ9000BT, (**E**) NZ9000T, (**F**) NZ9000TC and (**G**) NZ9000BC.

**Figure S2: Tricine-SDS-PAGE and Western blot analysis of *in vivo* secretion assay.**

The supernatants of NisA secreting *L. lactis* NZ9000 strains from different time points were TCA-precipitated and the pellets were analyzed by Tricine-SDS-PAGE and Western blot using an α-NisA_LP_ antibody (left panel). The Tricine-SDS-PAGE gels were stained by colloidal Coomassie (cc). The cytoplasmic and membrane fractions of the cell from different time points were analyzed by Western blot (right panel) with the specific antibodies (α-NisB, α-NisA_LP_, α-NisC and α-NBD). Sample analysis of *L. lactis* strains **A**) NZ9000BTC, (**B**) NZ9000BTC_H331A_, (**C**) NZ9000BT_H551A_C, (**D**) NZ9000BT, (**E**) NZ9000T, (**F**) NZ9000TC and (**G**) NZ9000BC.

M: marker protein bands

**Determination of NisT amount for NisA secretion rate**

**Figure S3: Determination of NisT amount of *in vivo* secretion assay samples.**

Calibration curves after Western blot analysis of membrane fractions from strains (**A**) NZ9000T (grey square) and (**B**) NZ9000BTC (red square) with an antibody raised against the NBD (α-NBD). Standard of known amount of T_NBD_ (black dots) was used to determine the amount of NisT (ng) in the membrane for time points 2-5 h. (**C)** A linear regression of plotted values of nmol NisA per nmol NisT against the time (min) results in a slope, which is the apparent secretion rate (V_s app_). Here, the molecular weight of NisT (~ 69 kDa; per monomer) and the correction factors, due to volume reduction at each time point (50 ml of the starting volume of 500 ml) were taken into account.

**Purification of NisA variants**

**Figure S4: Chromatograms of cIEX from uNisA, dNIsA and mNisA.**

The cIEX chromatograms and Tricine-SDS-PAGE of NisA variants (**A**) uNisA (grey), (B) dNisA (blue) and (C) mNisA (red). (**D**) The purity of the NisA leader peptide (NisA_LP_) was controlled by Tricine-SDS-PAGE. The Tricine-SDS-PAGE gels were stained by colloidal Coomassie (cc).

M: marker protein bands

**Pull-down assay**

**Figure S5: Western blot of pull-down assay with 10HNisT, NisB and NisC.**

Controls of the pull-down assay were blotted and analyzed with the specific antibodies of α-NisB, α-NBD and α-NisC. Samples of the sixth washing step (W_6_) and the elution fractions (E) were used to detect 10HNisT, NisB or NisC in the samples.

M: marker protein bands; +: addition of protein; -: no protein added

**Table supplemental information**

**Table S1: Strains and plasmids used in this study.**

| **strain** | **plasmid** | **name** | **properties** | **reference** |
| --- | --- | --- | --- | --- |
|  |  |  |  |  |
| *Escherichia coli* DH5α |  | DH5α | F– Φ80lacZΔM15 Δ(lacZYA-argF) U169 recA1 endA1 hsdR17 (rK–, mK+) phoA supE44 λ– thi-1 gyrA96 relA1 | ^10^ |
| *Lactococcus lactis* NZ9000 | - | NZ9000 | expression strain; *nisR* and *nisK* on the chromosome  (*pepN*::*nisRnisK*) | ^11^ |
| *L. lactis* NZ9000 | pIL-SV nisBTC  pNZ-SV nisA | NZ9000BTC | mNisA secreting strain | This study |
| *L. lactis* NZ9000 | pIL-SV nisBTC_H331A_  pNZ-SV nisA | NZ9000BTC_H331A_ | dNisA secreting strain | This study |
| *L. lactis* NZ9000 | pIL-SV nisBT_H551A_C  pNZ-SV nisA | NZ9000BT_H551A_C | non NisA secreting strain | This study |
| *L. lactis* NZ9000 | pIL-SV nisBT  pNZ-SV nisA | NZ9000BT | dNisA secreting strain | This study |
| *L. lactis* NZ9000 | pIL-SV nisT  pNZ-SV nisA | NZ9000T | uNisA secreting strain | This study |
| *L. lactis* NZ9000 | pIL-SV nisT_H551A_  pNZ-SV nisA | NZ9000T_H551A_ | non NisA secreting strain | This study |
| *L. lactis* NZ9000 | pIL-SV nisTC  pNZ-SV nisA | NZ9000TC | uNisA secreting strain | This study |
| *L. lactis* NZ9000 | pIL-SV nisBC  pNZ-SV nisA | NZ9000BC | non NisA secreting strain | This study |
|  |  |  |  |  |

**Table S2: MALDI-TOF-MS analysis of RP-HPLC fractions from *in vivo* secretion assay.**

The fractions of RP-HPLC were analyzed by MALDI-TOF-MS and are displayed as masses (M+H^+^) without or with start methionine. The modified peptides display eight or seven dehydrations and are expected for normally modified NisA by the nisin modification system.

| **peptide** | **sample** | **calc. mass**  **(Da)** | **observ. mass (+Met*)**  **(Da)** |
| --- | --- | --- | --- |
|  |  |  |  |
| mNisA | RP-HPLC run Fr. 33  from BTC+A supernatant | 5688  5706^#^ | 5689  5707^#^ |
| dNisA | RP-HPLC run Fr. 33  from BTC_H331A_+A supernatant | 5688  5706^#^ | 5689^#^ |
| dNisA | RP-HPLC run Fr. 33  from BT+A supernatant | 5688  5706^#^ | 5689  5707^#^ |
| uNisA | RP-HPLC run Fr. 31  From T+A supernatant | 5832 | 5834  (5951) |
| uNisA | RP-HPLC run Fr. 31  From TC+A supernatant | 5832 | n.d. |

\# 8x /7x dehydration

* plus start-methionine

n.d.: not determined

**Table S3: The determined kinetic parameter of the in vivo secretion assay.**

The supernatant of NisA secreting *L. lactis* strains was analyzed by RP-HPLC. The amount of NisA (nmol) plotted against time (min) was fitted with an allosteric sigmoidal fit and kinetic parameter were determined. Here, V_max_ is the maximal amount of secreted NisA, h is the Hill-coefficient and K_0.5_ is the time point, where half of the NisA is secreted. By determine the amount of NisT an apparent secretion rate (V_s app_) NisA•NisT^-1^•min^-1^ was calculated.

| **strain** | **V_max_ (nmol)** | **K_0.5_ (min)** | **h** | **V_s app_ (NisA•NisT^-1^•min^-1^)** |
| --- | --- | --- | --- | --- |
|  |  |  |  |  |
| NZ9000BTC | 534 ± 44 | 134 ± 12 | 2.9 ± 0.7 | 100.3 ± 35.2 |
| NZ9000BT_H551A_C | n.d. | n.d. | n.d. | n.d. |
| NZ9000BTC_H331A_ | 168 ± 16 | 200 ± 16 | 4.1 ± 0.9 | n.d. |
| NZ9000BT | 247 ± 15 | 152 ± 9 | 4.8 ± 1.2 | n.d. |
| NZ9000T | 137 ± 30 | 144 ± 41 | 1.9 ± 0.8 | 7.4 ± 1.6 |
| NZ9000TC | 38 ± 4 | n.d. | n.d. | n.d. |
| NZ9000BC | n.d. | n.d. | n.d. | n.d. |

n.d.: not determined

**Table S4: Oligonucleotides used in this study.**

The forward (for) and reverse (rev) oligonucleotides/primers used for the cloning are displayed in 5’-3’ direction. Mutated codons are underlined.

| **Name** | **Sequence (5’-3’)** |
| --- | --- |
|  |  |
| 10Hfor  10Hrev | CAAAATAAATTATAAGGAGGCAC  CGGATCTCAGTGGTA |
| infupNZ-SVfor  infupNZ-SVrev | CACCACCACCACCACTGAGATC  TGAGTGCCTCCTTATAATTTATTTTGTAG |
| infunisTfor  infunisTrev | TTTCAGGGCCCATGGATGGATGAAGTGAAAGAATTCA  GTGGTGGTGTCTAGATTATTCATCATTATCCTCATATTGC |
| linpNZ-SVfor  linpNZ-SVrev | TCTAGACACCACCACCACCACCACTG  CCATGGGCCCTGAAAATACAGGTTTTCGG |
| infupIL-SVfor  infupIL-SVrev | CGCGAGCATAATAAACGGCTCTGATTAAATTCTGAAGTTTG  CGTTTCAAGCCTTGGTTTTCTAATTTTGGTTCAAAGAAAG |
| Δ10Hfor | ATGGATGAAGTGAAAGAATTCACATCAAAAC |
| ΔnisT_TMD_for  ΔnisT_TMD_rev | CATATAGGAACTGTTAAAGTAATTAATTTATCATATG  CCATGGGCCCTGAAAATACAGGTTTTCGG |
| ΔnisTfor  ΔnisTrev | CTTTATTATTCAGAGCAATATGAGGATAATGATG  GTTTTGATGTGAATTCTTTCACTTCATCCATC |
| ΔnisBfor  ΔnisBrev | GACTAATAGATGGATGAAGTGAAAGAATTCAC  GTTTTTTCCTCTCTTTATTTTTATAAGCTATTTAGCAAC |
| ΔnisCfor  ΔnisCrev | GACCCAGCTTTCTTGTACAAAG  CCCATTGAGCAATAATTTTTTC |
| nisT_H551A__for  nisT_H551A__rev | CAATTTTCATTTCTGCAAGTTTGAATGCTG  CAGCATTCAAACTTGCAGAAATGAAAATTG |
| nisC_H331A_for  nisC_H331A_rev | CATATATGATTTGCGCAGGCTATTCTGGTTTAATAG CTATTAAACCAGAATAGCCTGCGCAAATCATATATG |
| termpNZfor  pnisArev | GAAAACCAAGGCTTGAAACG  CAGAGCCGTTTATTATGCTCGCG |
|  |  |

**Table S5: Additional plasmids used in this study.**

| **name** | **properties** | **reference** |
| --- | --- | --- |
| pNZ-SV | *E. coli* /*L. lactis* shuttle vector; ErmR; pnisA promoter; empty vector for cloning | ^12^ |
| pNZ-SVnisA | NisA expression | ^13^ |
| pNZ-SV10HnisT | NisT expression, deca-histidine tag at N-terminus; single alanine substitution at pos. H551 in the T_NBD_: ATP hydrolysis deficient mutant | This study |
| pNZ-SV10HnisT_H551A_ | NisT expression, deca-histidine tag at N-terminus | This study |
| pIL-SV | *E. coli* /*L. lactis* shuttle vector; CmR; pnisA promoter; empty vector for cloning | ^13^ |
| pIL-SVnisT | NisT expression | This study,  ^14^ |
| pIL-SVnisT_H551A_ | NisT expression; single alanine substitution at pos. H551 in the T_NBD_: ATP hydrolysis deficient mutant | This study |
| pIL-SVnisBTC | NisBTC expression | This study,  ^15^ |
| pIL-SVnisBTC_H331A_ | NisBTC expression; single alanine substitution at pos. H331 in the NisC; catalytically inactive NisC | This study, ^4^ |
| pIL-SVnisBT_H551A_C | NisBTC expression; single alanine substitution at pos. H551 in the T_NBD_: ATP hydrolysis deficient mutant | This study |
| pIL-SVnisBT | NisBT expression | This study,  ^14^ |
| pIL-SVnisTC | NisTC expression | This study,  ^14^ |
| pIL-SVnisBC | NisBC expression | This study,  ^14^ |
|  |  |  |
| pNGnisB6His | *L. lactis* expression vector; CmR; NisB expression; hexa-histidine tag and TEV-protease cleavage site at C-terminus | ^1,3^ |
| pET28b-AA-nisC | *E. coli* expression vector; KanR; NisC expression; hexa-histidine tag and thrombin cleavage site at N-terminus | ^2^ |

ErmR: erythromycin resistance; CmR: chloramphenicol resistance; KanR: kanamycin resistance ; T_NBD_: nucleotide binding domain of NisT

**References**

1	Mavaro, A. *et al.* Substrate recognition and specificity of the NisB protein, the lantibiotic dehydratase involved in nisin biosynthesis. *J Biol Chem* **286**, 30552-30560, doi:10.1074/jbc.M111.263210 (2011).

2	Abts, A., Montalban-Lopez, M., Kuipers, O. P., Smits, S. H. & Schmitt, L. NisC binds the FxLx motif of the nisin leader peptide. *Biochemistry* **52**, 5387-5395, doi:10.1021/bi4008116 (2013).

3	Reiners, J., Abts, A., Clemens, R., Smits, S. H. & Schmitt, L. Stoichiometry and structure of a lantibiotic maturation complex. *Sci Rep* **7**, 42163, doi:10.1038/srep42163 (2017).

4	Lubelski, J., Khusainov, R. & Kuipers, O. P. Directionality and coordination of dehydration and ring formation during biosynthesis of the lantibiotic nisin. *J Biol Chem* **284**, 25962-25972, doi:10.1074/jbc.M109.026690 (2009).

5	Laemmli, U. K. Cleavage of structural proteins during the assembly of the head of bacteriophage T4. *Nature* **227**, 680-685 (1970).

6	Schagger, H. Tricine-SDS-PAGE. *Nat. Protoc.* **1**, 16-22, doi:10.1038/nprot.2006.4 (2006).

7	Dyballa, N. & Metzger, S. Fast and sensitive colloidal coomassie G-250 staining for proteins in polyacrylamide gels. *J Vis Exp*, doi:10.3791/1431 (2009).

8	Schneider, C. A., Rasband, W. S. & Eliceiri, K. W. NIH Image to ImageJ: 25 years of image analysis. *Nat Methods* **9**, 671-675 (2012).

9	Lagedroste, M., Reiners, J., Smits, S. H. J. & Schmitt, L. Systematic characterization of position one variants within the lantibiotic nisin. *Sci Rep* **9**, 935, doi:10.1038/s41598-018-37532-4 (2019).

10	Grant, S. G., Jessee, J., Bloom, F. R. & Hanahan, D. Differential plasmid rescue from transgenic mouse DNAs into Escherichia coli methylation-restriction mutants. *Proceedings of the National Academy of Sciences of the United States of America* **87**, 4645-4649 (1990).

11	Kuipers, O. P., de Ruyter, P. G. G. A., Kleerebezem, M. & de Vos, W. M. Controlled overproduction of proteins by lactic acid bacteria. *Trends Biotechnol* **15**, 135-140, doi:10.1016/S0167-7799(97)01029-9 (1997).

12	AlKhatib, Z. *et al.* Lantibiotic immunity: inhibition of nisin mediated pore formation by NisI. *PLoS One* **9**, e102246, doi:10.1371/journal.pone.0102246 (2014).

13	AlKhatib, Z. *et al.* The C-terminus of nisin is important for the ABC transporter NisFEG to confer immunity in Lactococcus lactis. *Microbiologyopen* **3**, 752-763, doi:10.1002/mbo3.205 (2014).

14	van den Berg van Saparoea, H. B., Bakkes, P. J., Moll, G. N. & Driessen, A. J. Distinct contributions of the nisin biosynthesis enzymes NisB and NisC and transporter NisT to prenisin production by Lactococcus lactis. *Appl Environ Microbiol* **74**, 5541-5548, doi:10.1128/AEM.00342-08 (2008).

15	Rink, R. *et al.* Lantibiotic structures as guidelines for the design of peptides that can be modified by lantibiotic enzymes. *Biochemistry* **44**, 8873-8882, doi:10.1021/bi050081h (2005).

**Original Images of Figures**

**add Figure 3a: SDS-PAGE of purification of 10HNisT.**

**add Figure 3a: SDS-PAGE of purification of 10HNisTH551A.**

**add Figure 6a: Western blot pull-down experiment with Antibody against NisB.**

**add Figure 6a: Western blot pull-down experiment with Antibody against NisTNBD.**

**add Figure 6a: Western blot pull-down experiment with Antibody against NisTNBD.**

**add Figure 6a: Western blot pull-down experiment with Antibody against NisC.**

**add Figure 6b: Western blot pull-down experiment with Antibody against NisB.**

**add Figure 6b: Western blot pull-down experiment with Antibody against NisTNBD.**

**add Figure 6b: Western blot pull-down experiment with Antibody against NisC.**

**Original Images of Figures from SI**

**add Figure S2a: Tricine SDS-PAGE of TCA precipitated supernatants strain NZ9000BTC.**

**add Figure S2a: Western blot of TCA precipitated supernatants of strain NZ9000BTC with antibody against leader peptide.**

**add Figure S2a: Western blot cytoplasmic fraction of strain NZ9000BTC with antibody against NisB.**

**add Figure S2a: Western blot cytoplasmic fraction of strain NZ9000BTC with antibody against NisC.**

**add Figure S2a: Western blot cytoplasmic fraction of strain NZ9000BTC with antibody against leader peptide.**

**add Figure S2a: Western blot membrane fraction of strain NZ9000BTC with antibody against NisTNBD.**

**add Figure S2b: Tricine SDS-PAGE of TCA precipitated supernatants strain NZ9000BTCH331A.**

**add Figure S2b: Western blot of TCA precipitated supernatants of strain NZ9000BTCH331A with antibody against leader peptide.**

**add Figure S2b: Western blot cytoplasmic fraction of strain NZ9000BTCH331Awith antibody against NisB.**

**add Figure S2b: Western blot cytoplasmic fraction of strain NZ9000BTCH331Awith antibody against NisC.**

**add Figure S2b: Western blot cytoplasmic fraction of strain NZ9000BTCH331Awith antibody against leader peptide.**

**add Figure S2b: Western blot membrane fraction of strain NZ9000BTCH331A with antibody against NisTNBD.**

**add Figure S2c: Tricine SDS-PAGE of TCA precipitated supernatants strain NZ9000BTH551AC.**

**add Figure S2c: Western blot of TCA precipitated supernatants strain NZ9000BTH551AC with antibody against leader peptide.**

**add Figure S2c: Western blot cytoplasmic fraction of strain NZ9000BTCH331Awith antibody against NisB.**

**add Figure S2c: Western blot cytoplasmic fraction of strain NZ9000BTCH331Awith antibody against NisC.**

**add Figure S2c: Western blot cytoplasmic fraction of strain NZ9000BTCH331Awith antibody against leader peptide**

**add Figure S2c: Western blot membrane fraction of strain NZ9000BTCH331A with antibody against NisTNBD.**

**add Figure S2d: Tricine SDS-PAGE of TCA precipitated supernatants strain NZ9000BT.**

**add Figure S2d: Western blot of TCA precipitated supernatants strain NZ9000BT with antibody against leader peptide.**

**add Figure S2d: Western blot cytoplasmic fraction of strain NZ9000BTwith antibody against NisB.**

**add Figure S2d: Western blot cytoplasmic fraction of strain NZ9000BTwith antibody against leader peptide.**

**add Figure S2d: Western blot membrane fraction of strain NZ9000BT with antibody against NisTNBD.**

**add Figure S2e: Tricine SDS-PAGE of TCA precipitated supernatants strain NZ9000T.**

**add Figure S2e: Western blot of TCA precipitated supernatants strain NZ9000T with antibody against leader peptide.**

**add Figure S2e: Western blot cytoplasmic fraction of strain NZ9000T with antibody against leader peptide.**

**add Figure S2e: Western blot membrane fraction of strain NZ9000T with antibody against NisTNBD.**

**add Figure S2f: Tricine SDS-PAGE of TCA precipitated supernatants strain NZ9000TC.**

**add Figure S2f: Western blot of TCA precipitated supernatants strain NZ9000TC with antibody against leader peptide**

**add Figure S2e: Western blot cytoplasmic fraction of strain NZ9000TC with antibody against NisC.**

**add Figure S2e: Western blot cytoplasmic fraction of strain NZ9000TC with antibody against leader peptide.**

**add Figure S2f: Western blot membrane fraction of strain NZ9000TC with antibody against NisTNBD.**

**add Figure S2g: Tricine SDS-PAGE of TCA precipitated supernatants strain NZ9000BC.**

**add Figure S2g: Western blot of TCA precipitated supernatants strain NZ9000BC with antibody against leader peptide**

**add Figure S2g: Western blot cytoplasmic fraction of strain NZ9000BC with antibody against NisB.**

**add Figure S2g: Western blot cytoplasmic fraction of strain NZ9000BC with antibody against NisC.**

**add Figure S2g: Western blot cytoplasmic fraction of strain NZ9000BC with antibody against leader peptide.**

**add Figure S4a: Tricine SDS-PAGE of IEX purification of uNisA from NZ9000T supernatant**

**add Figure S4b: Tricine SDS-PAGE of IEX purification of dNisA from NZ9000BT supernatant**

**add Figure S4c: Tricine SDS-PAGE of IEX purification of mNisA from NZ9000BTC supernatant**

**add Figure S4d: Tricine SDS-PAGE of NisA leader peptide synthesized by JPT peptide technologies**

**add Figure S5: Western blot of pull-down assay controls with antibody against NisB.**

**add Figure S5: Western blot of pull-down assay controls with antibody against NisTNBD.**

**add Figure S5: Western blot of pull-down assay controls with antibody against NisC**
